# The role of mitochondria-related genes and immune infiltration in carotid atherosclerosis: identification of hub targets through bioinformatics and machine learning approaches

**DOI:** 10.3389/fgene.2025.1597445

**Published:** 2025-08-05

**Authors:** Dan Liu, Kun Guo, Min Li, Xiaochen Yu, Xue Guan, Xiuru Guan

**Affiliations:** Department of Laboratory Diagnostics, First Affiliated Hospital of Harbin Medical University, Harbin, Heilongjiang, China

**Keywords:** atherosclerosis, bioinformatics analysis, carotid artery, hub Mito-DEGs, immune infiltration, mitochondria

## Abstract

**Objective:**

Atherosclerosis (AS) is the underlying pathology of atherosclerotic cardiovascular disease and a major cause of cardiovascular-related mortality. Chronic inflammation and mitochondrial dysfunction, triggered by the infiltration of various immune cells, are key factors in the progression of AS. However, the interaction and crosstalk between these factors remain unclear.

**Methods:**

Two gene expression datasets, GSE100927 and GSE43292, were downloaded from the National Center for Biotechnology Information Gene Expression Omnibus (NCBI GEO) database, covering carotid atherosclerosis and control groups. After identifying the common differentially expressed genes (DEGs), mitochondria-related DEGs (Mito-DEGs) were obtained through Weighted Gene Co-expression Network Analysis (WGCNA) and machine learning approaches. Immune infiltration analysis and comparison were subsequently performed. The single-cell dataset GSE159677 further validated the expression proportions of relevant genes in different cell populations during the progression of AS. Additionally, cell-cell communication and trajectory analysis within the immune landscape were utilized to infer the pathways of cell state transitions within AS clusters. THP-1 cells were cultured *in vitro*, and the foam cell model was established by the addition of oxidized low-density lipoprotein (ox-LDL). The expression trends of hub Mito-DEGs were confirmed via qRT-PCR.

**Results:**

From the GSE100927 and GSE43292 datasets and the MitoCarta3.0 database, three hub Mito-DEGs closely associated with AS were ultimately identified: *CASP8*, *GATM*, and *LAP3*. Subsequent comprehensive bioinformatics analysis of these hub genes further emphasized the importance of the immune system in AS. Immune profiling based on the CIBERSORT algorithm revealed significantly increased infiltration of activated mast cells, monocytes, memory B cells, T follicular helper cells, and M0 macrophages in the immune microenvironment of AS. Single-cell analysis showed that *GATM* and *LAP3* were enriched in monocytes and macrophages, while *CASP8* exhibited increased expression in NK cells, T cells, and monocytes. In addition, *in vitro* cell experiments demonstrated that mRNA expression levels of the hub Mito-DEGs were significantly elevated in the lipid-laden foam cell group compared to the control group, consistent with the expression patterns observed in the single-cell dataset.

**Conclusion:**

This study revealed the interaction between Mito-DEGs and the immune system in AS. These findings may provide new insights into therapeutic monitoring and prognosis evaluation.

## 1 Introduction

Atherosclerotic cardiovascular disease (ASCVD), which incorporates ischaemic stroke and ischaemic heart disease (IHD) (Zhao et al., 2019), is a prominent contributor to disability-adjusted life-years and premature death on a global scale (Reiner et al., 2011; GBD, 2019 Stroke Collaborators, 2021). Atherosclerosis (AS), an important factor in the development of cardiovascular disease (CVD), is a chronic inflammatory disease (Bjorkegren and Lusis, 2022). It is characterized by intimal plaque formation, cholesterol accumulation in the arterial walls, endothelial dysfunction, and the generation of pro-inflammatory cytokines (Hennekens and Gaziano, 1993; Badimon et al., 2011; Goodman et al., 2015). The progression of AS is accelerated by the involvement of the adaptive immune system (Khan et al., 2024). Recent studies have found that theranostic photoactivation technology can simultaneously assess plaques and promote inflammation resolution in the inflammatory process underlying AS (Kim et al., 2024). It has been demonstrated that PCSK9 inhibitors have the ability to reduce low-density lipoprotein (LDL) cholesterol, which is used to treat dyslipidemia and slow the progression of AS (Gennemark et al., 2021). Despite advances in targeting plaque inflammation and lipid deposition from the etiological perspective of AS, studies have indicated that global mortality from IHD and ischaemic stroke has continued to rise over the past 20–30 years, reaching 15.69 million deaths in 2019, accounting for about 84% of all CVD-related deaths (Roth et al., 2020). The clinical outcomes and prognosis of IHD and ischaemic stroke remain concerning. Therefore, there is an urgent need for validated biomarkers as early screening tools or potential therapeutic targets.

Changes in vascular cell function often occur before the onset of cardiovascular disease. Mitochondria, through coordination with other organelles, play a critical role in determining cell function (Beg et al., 2024). Recent research has shown that mitochondria are involved in nearly all aspects of cell biology, including cell differentiation, inflammation, autophagy, innate immunity, programmed cell death, redox signaling, calcium homeostasis, and lipid metabolism (Smith et al., 2012; Murphy and Hartley, 2018; Chan, 2020; Giacomello et al., 2020; Raghu et al., 2021; Shi et al., 2024). Due to the central role of mitochondria in cellular function, many common diseases, even when not primarily caused by mitochondrial dysfunction, may exhibit “secondary” mitochondrial disorders, such as metabolic, cardiovascular, neurodegenerative, and neuromuscular diseases (Whitaker et al., 2016; Herzig and Shaw, 2018; Sorrentino et al., 2018; Suomalainen and Battersby, 2018; Shi et al., 2024). A recent study by *Zheng et al.* (Zheng et al., 2025) demonstrated that methyltransferase-like protein 4 (METTL4)-mediated N6-methyldeoxyadenosine (6 mA) modification of mitochondrial DNA (mtDNA) induces mitochondrial damage and inflammatory responses in macrophages, thereby promoting the development of atherosclerosis. Therefore, integrating the transcriptomic characteristics of AS with mitochondrial function may offer new perspectives in this field.

The objective of this study was to identify the correlation between AS and mitochondria-related genes utilizing data obtained from the Gene Expression Omnibus (GEO) database. Hub genes were identified by the intersection of differentially expressed genes (DEGs), weighted gene co-expression network analysis (WGCNA), and mitochondrial-related gene sets. In addition, various machine learning (ML) methods were employed to identify key biomarkers and assess their diagnostic value in AS. The percentage of immune cell infiltration linked to these biomarkers was then ascertained. Finally, single-cell analysis was adopted to identify the distribution of key biomarkers, followed by validation through *in vitro* analyses. By finding potential biomarkers for risk assessment in AS patients, this study aims to shed light on the basic mechanisms underlying the disease.

## 2 Materials and methods

### 2.1 Data collection and preprocessing

From the NCBI GEO public database (https://www.ncbi.nlm.nih.gov/geo/), the AS datasets were acquired. The GSE100927 dataset, annotated via the GPL17077 platform (Agilent), encompasses RNA expression profiles from 104 human arterial samples, including carotid, femoral, and infra-popliteal arteries. For this study, carotid artery samples (most suitable for early diagnosis of atherosclerosis) were selected for analysis, consisting of 29 human carotid atherosclerotic samples and 12 carotid artery control samples. The GSE43292 dataset was selected to validate the hub genes. The dataset is annotated using the GPL6244 platform (Affymetrix) and includes 32 carotid atherosclerosis samples and 32 carotid artery control samples from humans (Table 1). In order to standardize the raw data from these datasets, CEL files were imported using the “Affy” package, with background correction performed via Robust Multi-Array Average (RMA), followed by log2 transformation for normalization. The R program “limma” was employed for subsequent analysis. In the Affymetrix dataset, 22 mitochondrial genes were detected (Supplementary Material S1). A comprehensive set of 1,136 mitochondria-related genes was retrieved from the MitoCarta3.0 database at http://www.broadinstitute.org/mitocarta.

**TABLE 1:** Baseline information for three data sets in this study.

GEO	GPL	Sample	N	Type
GSE100927	GPL17077 Agilent-039494 SurePrint G3 Human GE v2 8 × 60K Microarray 039381	AS/Control	69/35	Microarray
GSE43292	GPL6244 Affymetrix Human Gene 1.0 ST Array	AS/Control	32/32	Microarray
GSE159677	GPL18573 Illumina NextSeq 500	AC/PC	3/3	single cell RNA-seq

### 2.2 Analysis of DEGs

With the “limma” package (3.60.4) in R, DEGs between the AS samples and normal controls were identified (Ritchie et al., 2015), with adjusted P < 0.05 and fold change (FC) > 1.3 as the criteria. To visualize the DEGs, hierarchical clustering heatmaps and volcano plots were generated by the “ggplot2” package (3.5.1). To identify common DEGs between the GSE100927 and GSE43292 datasets, the “VennDiagram” package (1.7.3) was adopted, with the results described accordingly.

### 2.3 Functional enrichment analysis via GO and KEGG

Based on the official websites of the GO and KEGG databases (at http://geneontology.org/, https://www.genome.jp/KEGG/, respectively) (Kanehisa and Goto, 2000), the R packages “enrichplot” (1.24.2), “clusterProfiler” (4.12.1) (Yu et al., 2012), and “org.Hs.eg.db” (3.19.1) were utilized to analyze the significant functions and pathways of the DEGs. DEGs screened from the GSE100927 and GSE43292 datasets were first mapped from gene symbols to Entrez IDs using the “org.Hs.eg.db” package, with unmapped genes excluded to ensure that all genes included in subsequent analyses had valid annotations. All DEGs were included in the analysis, and the background gene set was defined as the complete human genome annotation provided by org.Hs.eg.db. Gene functions were evaluated using Gene Ontology (GO) analysis, covering biological process (BP), cellular component (CC), and molecular function (MF). Enrichment analysis was performed using the enrichGO function, and enriched terms with P < 0.05 were retained. Significant pathways were ultimately visualized using bubble plots. To identify signaling pathways, KEGG analysis was conducted using the enrichKEGG function, restricted to human pathways. Pathways were selected based on an adjusted *P* < 0.05 as the cutoff. The enrichment results for significantly enriched pathways were visualized using bubble plots generated by the “ggplot2” package (version 3.5.1).

### 2.4 Identification of significant modules based on WGCNA

Co-expression modules linked to AS were identified using WGCNA analysis with the “WGCNA” package (1.72–5) in R (Langfelder and Horvath, 2008). The top 25% of genes showing the highest variance in the dataset were chosen (Chen S. et al., 2020; Feng S. et al., 2022), and the pickSoftThreshold function was used to calculate the optimal soft-threshold power (β), followed by the adjacency matrix transformation. Next, the topological overlap matrix (TOM) was computed. A hierarchical clustering dendrogram was then generated to segment genes exhibiting comparable expression patterns into separate modules, with a minModuleSize of 30. A dynamic tree-cutting method was applied to identify modules of highly correlated genes. At last, module eigengenes (MEs) were used to consolidate the expression profiles of each module, allowing the computation of the correlation between MEs and clinical traits. The most pertinent modules obtained were chosen for additional analysis (Rezaei et al., 2022; Gao et al., 2023; Wang et al., 2023b; Wang Q. et al., 2024).

### 2.5 Feature gene screening

To identify hub Mito-DEGs in AS, three algorithms were employed: least absolute shrinkage and selection operator (LASSO), random forest (RF), and support vector machine-recursive feature elimination (SVM-RFE) (Qin et al., 2023; Qin et al., 2024). LASSO is a commonly used data mining method for multivariate linear regression (Bai et al., 2022), and LASSO regression analysis was conducted by the package “glmnet” (4.1–8). LASSO improves both the predictive performance and interpretability of statistical models by incorporating a regularization term into the loss function, allowing for feature screening. This enables feature selection by shrinking coefficient estimates towards zero, with the regularization parameter λ determining the extent of shrinkage. The optimal values of λ and γ were obtained through 10-fold cross-validation to prevent model overfitting (Kang et al., 2021; Liu et al., 2024). SVM-RFE is a linear classifier used for the binary classification of data through supervised learning (Li et al., 2024). RF, an algorithm provided by the “randomForest” package (4.7–1.1), is a randomized method designed to prevent the overfitting of individual decision trees and enhance the model performance built from multiple correlated decision trees obtained from the identical training set (Guan et al., 2024). We initially set the number of trees to ntree = 500 to generate a random forest. The optimal number of trees was identified based on the smallest cross-validation error, and gene importance was then ranked. Genes that had an importance score greater than 1 were selected for further analysis. After that, the intersection of results from the three algorithms was determined, and a Venn diagram was generated with “VennDiagram” (1.7.3) in R. The packages “pROC” (1.18.5) and “InpROC” function were selected to draw receiver operating characteristic (ROC) curves and to compute the corresponding area under the curve (AUC) to find out the predictive performance of these feature genes in both the validation and training sets. The “rms” (6.8–1) and “rmda” (1.6) packages were used to generate a nomogram based on the identified feature genes. After that, a calibration curve was created to evaluate the nomogram’s accuracy. Also, the clinical impact curve of the model was graphed and assessed. Finally, the clinical utility of the nomogram was assessed with the help of decision curve analysis.

### 2.6 Analysis of the relationship between Mito-DEGs and immune cells

The relative abundance of each immune cell subtype was determined based on the LM22 reference matrix of the CIBERSORT algorithm, using sequencing data from human carotid artery samples with or without atherosclerosis (Newman et al., 2015). The aforementioned subtypes correspond to the cellular makeup of the immunological microenvironment. A p-value was then calculated by CIBERSORT with Monte Carlo sampling for sample deconvolution, which indicated the confidence level in the results. Samples with estimated immune cell fractions were considered accurate if P < 0.05 (Chen et al., 2023; Pan et al., 2023). Comparative analysis of immune cell populations among groups was conducted using the Wilcoxon test. The Spearman correlation coefficient was then applied to ascertain the connection between immune cells and hub genes. The findings were then graphically represented using a lollipop plot (Li and Cai, 2024).

### 2.7 Single-cell analysis

The GSE159677 dataset was acquired to conduct additional studies on model genes (Alsaigh et al., 2022). The package “Seurat” (5.1.0) (Gribov et al., 2010) was utilized to analyze the scRNA-seq data. Low-quality cells were eliminated by applying specific criteria: genes were required to be expressed in at least three cells, the number of genes detected per cell was limited to between 500 and 8,000, and the proportion of mitochondrial genes was set at less than 20%. For principal component analysis (PCA), we selected the top 8,000 genes with the highest expression variability. The merge function was used to integrate single-cell data, and the NormalizeData function was applied for normalization. Then, t-distributed stochastic neighbor embedding (t-SNE) and principal component analysis were applied. Uniform manifold approximation and projection (UMAP) was adopted for dimension reduction and cell cluster identification. The cell types within various clusters were annotated using the “SingleR” in R (Pan et al., 2023; Yang et al., 2023). At last, to find marker genes for every cell cluster, the FindAllMarkers was utilized. After that, incorporated cell-cell communication analysis and pseudotime trajectory analysis. Monocle 3 was used with UMAP/v0.3.2 to project the data into a low-dimensional space (Cao et al., 2019; Jean-Baptiste et al., 2019) to enable pseudotime trajectory analysis. Cell-cell communication analysis was performed using the R package CellChat (Jin et al., 2021).

### 2.8 Cell culture

Cell culture was performed using the THP-1 cell line, which was kindly provided by Wuhan Pricella Biotechnology Co., Ltd. THP-1 cells were cultured in RPMI 1640 (Gibco, United States) medium supplemented with 10% heat-inactivated fetal bovine serum (FBS, FSP500, Excell, China) (incubation conditions: 5% CO_2_, 37°C). Differentiation into macrophages was induced by treating THP-1 cells with 100 ng/mL phorbol 12-myristate 13-acetate (PMA, MCE, United States) for 48 h. Following induction, cells were exposed to oxidized low-density lipoprotein (ox-LDL, YB-002, Yiyuan Biotechnologies, China) at a concentration of 50 μg/mL and cultured for 48 h (foam group), or maintained in complete medium for 48 h (control group).

### 2.9 *In vitro* generation of foam cells

THP-1 cells were seeded onto cell climbing slides and differentiated into macrophages by treatment with PMA for 48 h, followed by stimulation with ox-LDL to induce transformation into foam cells. To analyze lipid droplet content in THP-1 cells, BODIPY 493/503 (C2053S, Beyotime Institute of Biotechnology, Jiangsu, China) staining was performed: cells were incubated with the dye for 30 min, then the stained cell climbing slides were mounted on coverslips with mounting medium. Fluorescence microscopy (EUROStar III Plus) was used for observation, images were captured with the EUROPattern II fluorescence imaging system, and fluorescence intensity was quantitatively measured using Image.

### 2.10 RNA extraction and qRT-PCR

Total RNA from the two groups of cells was extracted from cultured cells with RNAiso Plus from TaKaRa (9109, Beijing, China). The extracted RNA was reverse transcribed into cDNA with the 5× PrimeScript™ RT Master Mix from TaKaRa (RR036A, Beijing, China). Subsequently, qPCR was carried out with 2× SYBR Green qPCR MasterMix II (Sevenbio, SM143-01, Beijing, China). For subsequent mRNA qPCR, *GAPDH* served as the internal control, and target gene expression was quantified relative to *GAPDH* with the 2^−ΔΔCT^ method. Statistical analysis of differences was performed using independent sample t-tests, with statistical significance set at P < 0.05. The gene primer sequences identified are provided in Table 2.

**TABLE 2:** Primer sequences of *homo sapiens* genes by RT-qPCR.

Target genes	Primer sequences
*GAPDH*	5′-GAG​TCA​ACG​GAT​TTG​GTC​GT-3′ (forward)
	5′-GAC​AAG​CTT​CCC​GTT​CTC​AG-3′ (reverse)
*CASP8*	5′-GCT​GAC​TTT​CTG​CTG​GGG​AT-3′ (forward)
	5′-GAC​ATC​GCT​CTC​TCA​GGC​TC-3′ (reverse)
*GATM*	5′-GAC​AAA​GCC​ACT​GAG​CCT​CT-3′ (forward)
	5′-CTC​GAT​GGT​GAA​CGG​TGG​AA-3′ (reverse)
*LAP3*	5′-AAG​CCG​GGG​GAT​GTT​GTT​AG-3′ (forward)
	5′-AGT​GGC​ACC​TGA​TCC​CAA​AG-3′ (reverse)

### 2.11 Mitochondrial expression in foam cells

Following the aforementioned culture and treatment protocols, both control and foam groups were incubated with Mito-Tracker Green (C1048, Beyotime Institute of Biotechnology, Nantong, China) at 37°C for 30 min and subsequently analyzed using a FACSLyric flow cytometer (BD Biosciences).

### 2.12 Statistical analysis

For statistical analysis, R (4.4.1) and GraphPad Prism 9 were utilized, including “limma”, “ggplot2”, “VennDiagram”, “clusterProfiler”, “WGCNA”, “glmnet”, “randomForest”, “VennDiagram”, “pROC”, “rms”, and “rmda”. To determine the significance of differences, independent sample t-tests were utilized, and data were expressed as mean ± standard deviation. Statistics were considered significant when *P* < 0.05.

## 3 Results

### 3.1 DEGs in AS and functional enrichment analysis

**FIGURE 1:**
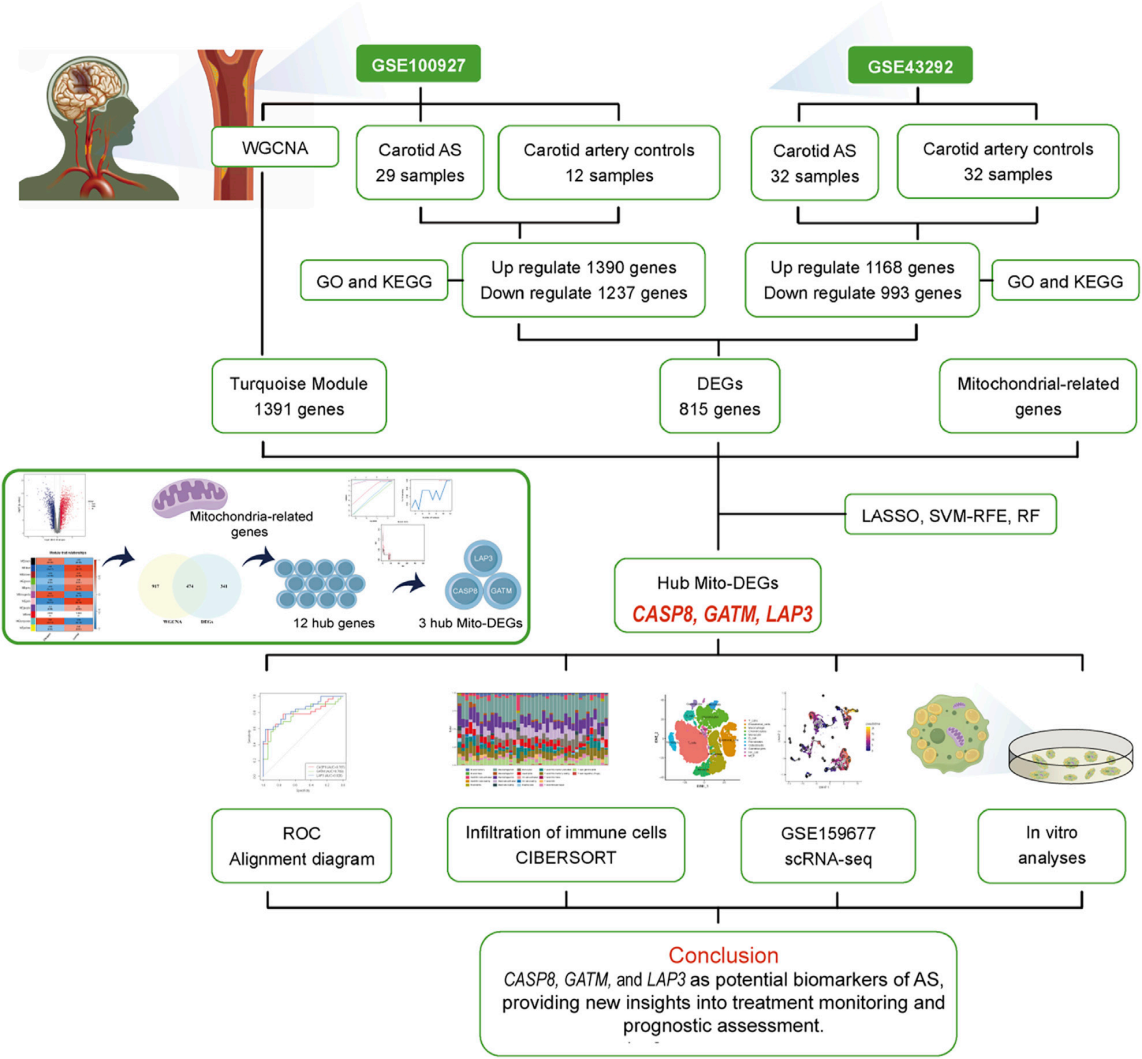
Workflow of strategies targeting mitochondria-related hub genes in AS.

**FIGURE 2:**
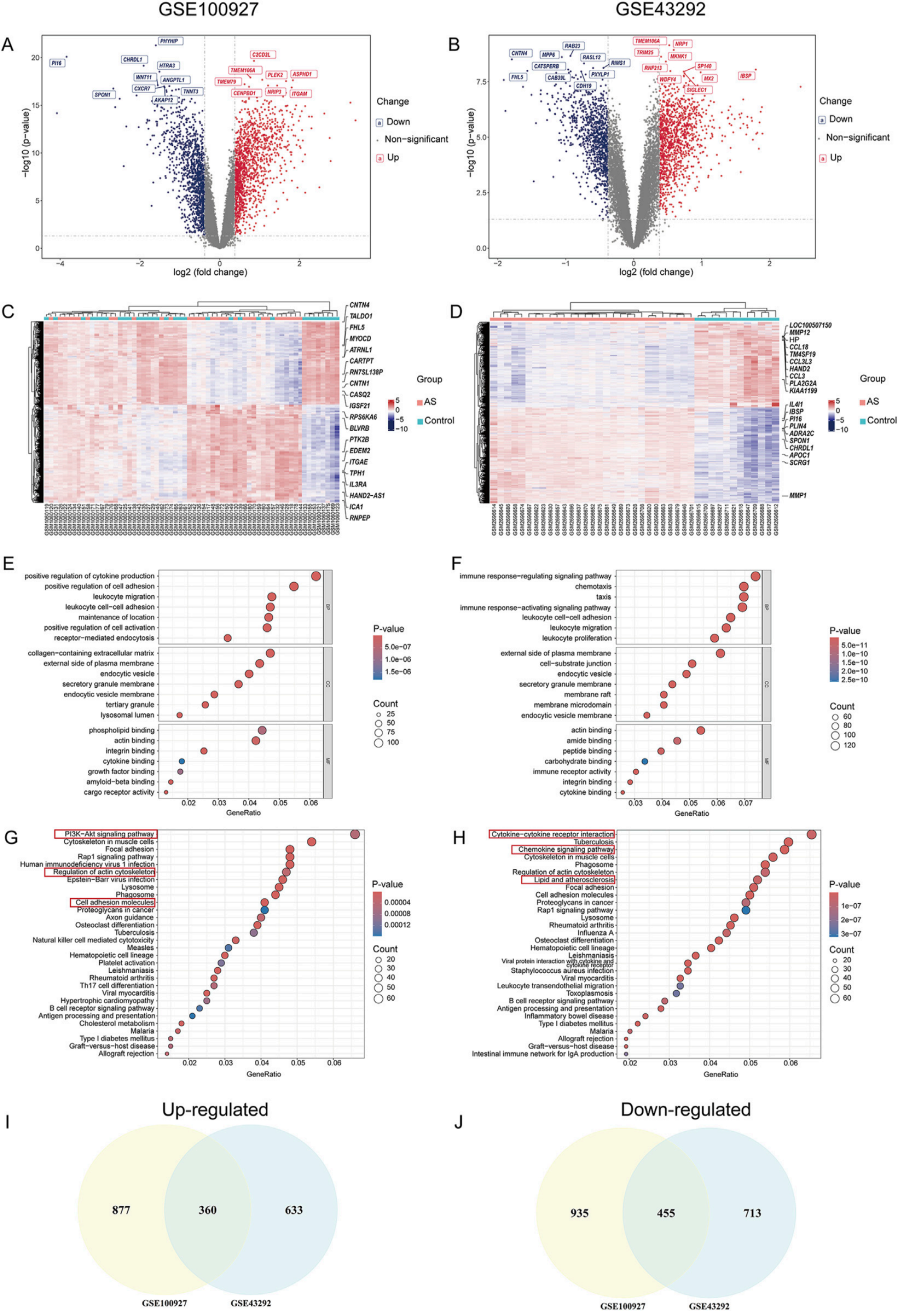
Results of DEG and GO-KEGG Analysis in AS. **(A,B)** Volcano plots of DEGs in GSE100927 and GSE43292; **(C,D)** Heatmaps of DEG clustering for GSE100927 and GSE43292; **(E,F)** Enriched GO terms for DEGs in GSE100927 and GSE43292; **(G,H)** KEGG pathway enrichment results for GSE100927 and GSE43292; **(I)** Venn diagram of upregulated DEGs in the GSE100927 and GSE43292 datasets; **(J)** Venn diagram of downregulated DEGs in the GSE100927 and GSE43292 datasets.

The overall workflow of the study is illustrated in Figure 1. Two AS-related GEO datasets, GSE100927 (including 41 samples) and GSE43292 (including 64 samples), were analyzed (Supplementary Figure S1). Differential expression analysis revealed 1,390 genes upregulated and 1,237 genes downregulated in carotid plaque samples from the GSE100927 dataset, while the GSE43292 dataset included 1,168 upregulated and 993 downregulated genes. Heatmaps and volcano plots were used to display the DEGs (Figures 2A–D). Functional enrichment analysis of DEGs was subsequently performed using GO and KEGG pathway analyses. GO analysis, encompassing BP, CC, and MF terms, showed major enrichments in cell adhesion, the external side of the plasma membrane, actin binding, integrin binding, and cytokine binding (Figures 2E,F) (Supplementary Figure S2). Significant enrichment was found in the PI3K−Akt signaling pathway, immune-related pathways, and lipid and atherosclerosis pathways, according to KEGG pathway analysis (Figures 2G,H). Recognizing that genes exhibiting distinct trends under varying regulatory conditions may hold distinct biological implications, we performed GO and KEGG pathway analyses separately for the upregulated and downregulated gene sets derived from two distinct datasets. It was found that upregulated genes are significantly enriched in functions related to cytokine production and immune responses. Furthermore, these genes play crucial roles in pathways including Lysosome, Phagosome, and lipid-related processes. In contrast, downregulated genes are enriched in pathways associated with muscle and focal adhesion (Supplementary Figures S2, S3). After intersecting the DEGs from the two datasets, 815 common DEGs were identified (Figures 2I,J).

### 3.2 WGCNA

**FIGURE 3:**
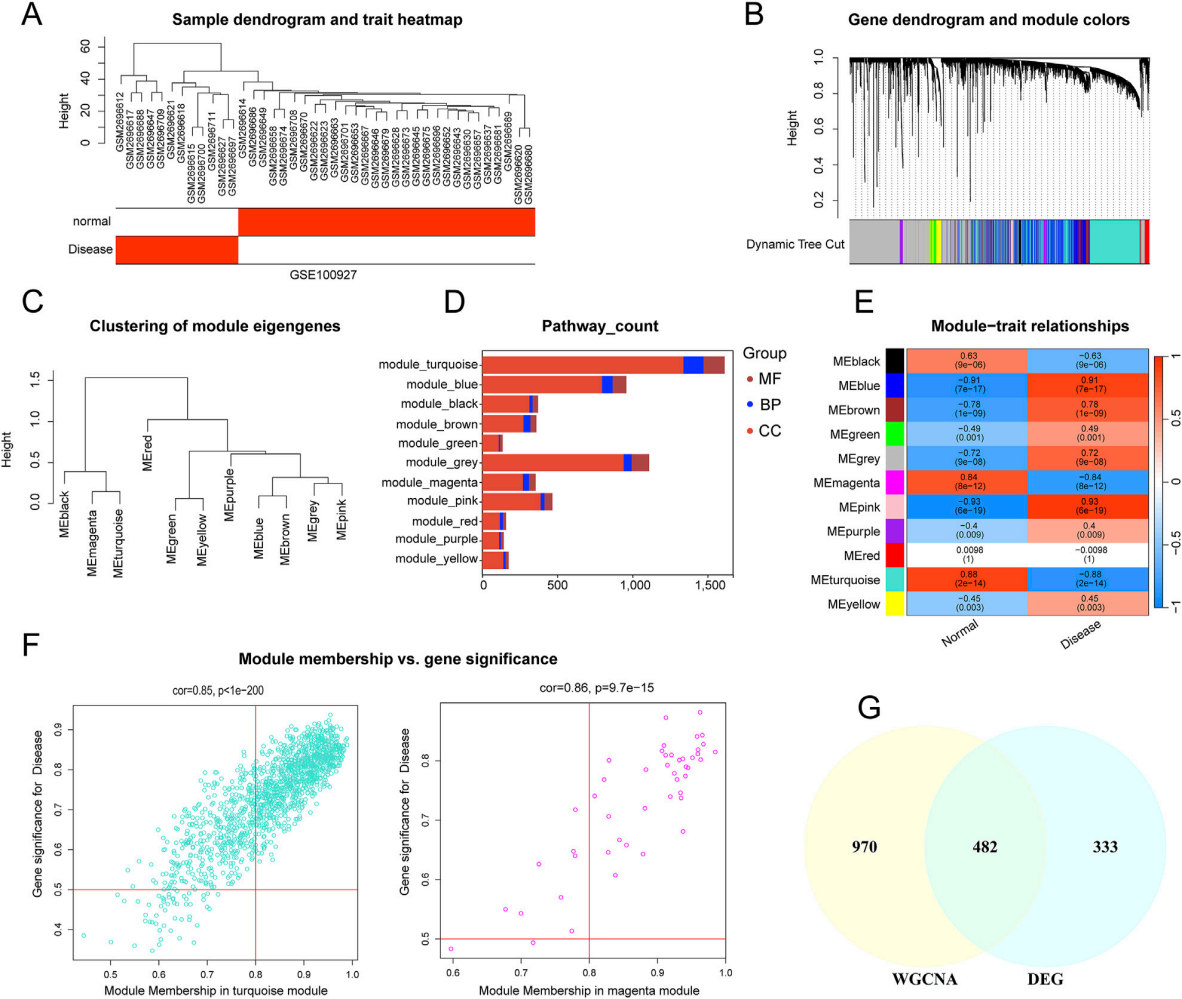
Construction of Weight Gene Co-Expression Network and Identification of the Key Module. **(A)** Sample dendrogram and trait heatmap; **(B)** Hierarchical clustering dendrogram of co-expression modules, with different colors representing distinct modules. **(C)** Hierarchical clustering of eigengenes summarizing the identified modules in the clustering analysis. **(D)** Stacked box plots of pathway-count for each module **(E)** WGCNA module-trait relationship heatmap showing correlations between modules and traits. The turquoise and pink modules are highly correlated with atherosclerosis. **(F)** Scatterplot showing the relationship between module membership in the two modules and gene significance (turquoise and magenta). **(G)** Venn diagram illustrating the overlap between genes in two modules highly correlated with AS in WGCNA and DEGs.

This study employed WGCNA to identify module genes linked to carotid AS. First, the top 25% of genes with the highest expression variability were selected from 32 carotid atherosclerotic plaque samples and 19 control samples for hierarchical clustering analysis. Euclidean distance was used to calculate similarity between samples, and average linkage was applied to construct the clustering dendrogram (Figure 3A). To achieve high connectivity and scale independence among genes within each module, the optimal soft-threshold power was set to 20, forming a scale-free co-expression network (Supplementary Figure S4). Eleven co-expression modules of various colors were created by hierarchical clustering of the samples using a dynamic tree-cutting algorithm. (Figures 3B,C). The composition of pathway-related genes among different modules was analyzed (Supplementary Figure S3). Most pathways in the purple and grey modules were primarily associated with cellular processes, including cell differentiation, regulation of activation, cell adhesion, and responses to various stimuli (such as positive regulation of T cell differentiation, positive regulation of cell activation, positive regulation of cell–cell adhesion, and cellular response to hypoxia). In contrast, the yellow module was predominantly linked to developmental processes (such as pathways related to kidney development), the black module was mainly enriched in immune-related pathways (such as lymphocyte differentiation and regulation of T cell activation), and the green module was largely associated with metabolic processes (such as aldosterone biosynthetic process). The GO terms for each module were visualized as stacked box plots (Figure 3D). Next, we analyzed the co-expression similarity and adjacency of modules, and their associations with clinical traits (carotid control and AS groups). Finally, the turquoise module, which showed the strongest correlation with AS, was identified (Figure 3E), containing 1,391 genes (Figure 3F). The magenta module, comprising 61 genes, showed a very similar correlation coefficient (0.88 for turquoise versus 0.84 for magenta). Therefore, genes from these two most significant modules were combined and intersected with the 815 DEGs, resulting in the identification of 482 overlapping genes (Figure 3G).

### 3.3 Identification of Mito-DEGs in AS and feature gene screening

**FIGURE 4:**
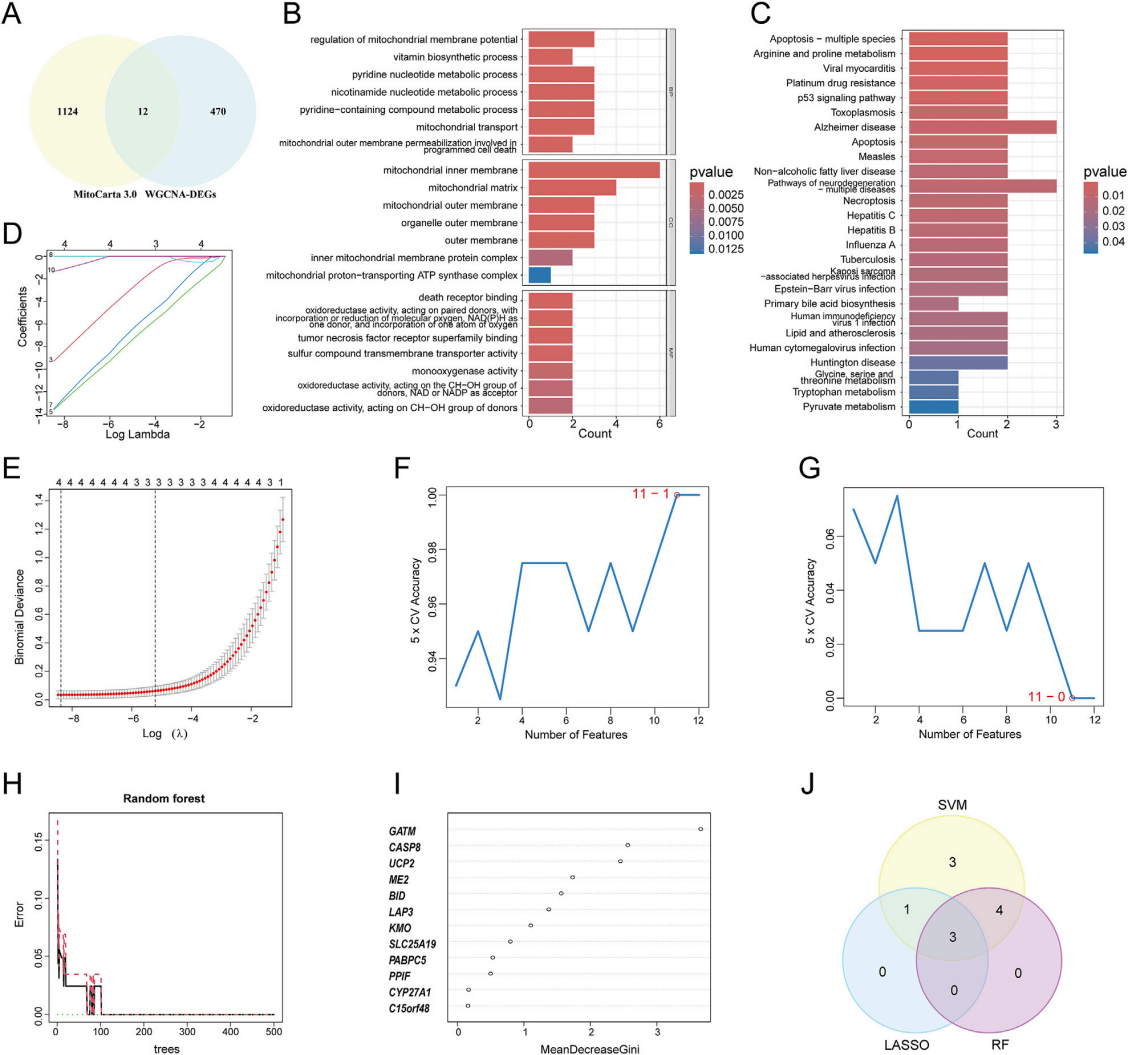
The final hub MitoDEGs were identified using three machine learning (ML) algorithms. **(A)** Venn diagram showing the overlap between genes in WGCNA-DEGs and MitoCarta3.0; **(B,C)** GO and KEGG enrichment analyses of mitochondria-related DEGs; **(D)** LASSO coefficient path diagram, where each curve represents a single gene; **(E)** Lasso regression cross-validation curve. The optimal λ values were determined using 10-fold cross-validation, identifying a total of 4 hub genes; **(F)** The SVM-RFE algorithm identified the highest accuracy when using 11 genes; **(G)** The SVM-RFE algorithm determined the lowest error rate when using 11 genes; **(H)** The relationship between the number of Random Forest trees and the error rate; **(I)** Genes ranked in descending order of importance; **(J)** Venn diagrams showing the overlap of genes identified by the three algorithms.

As a central regulator of metabolism, mitochondria play a pivotal role in cellular energy metabolism, stress response, and the maintenance of internal homeostasis, thereby governing cell fate (Harrington et al., 2023). In recent years, targeting mitochondrial dysfunction has emerged as a novel therapeutic strategy for the prevention and treatment of atherosclerotic cardiovascular diseases, yielding significant progress (Bravo-San Pedro et al., 2017; Becker et al., 2023; Zheng et al., 2025). To systematically and scientifically screen for potentially actionable mitochondria-related genes in atherosclerosis, a total of 1,136 mitochondria-related genes were selected from the MitoCarta3.0 database. The intersection of WGCNA-derived DEGs and these mitochondria-related genes was obtained. The overlapping genes were deemed as mitochondria-related DEGs (Figure 4A). Twelve mitochondria-related DEGs were identified, including *PPIF, SLC25A19, ME2, GATM, LAP3, CYP27A1, KMO, UCP2, BID, C15orf48, CASP8, and PABPC5*. These DEGs were enriched in BP terms such as regulation of mitochondrial membrane potential, in CC for the mitochondrial inner membrane, and in MF for death receptor binding and oxidoreductase activity (Figure 4B). KEGG pathway analysis suggested enrichment of these genes in apoptosis, arginine and proline metabolism, and Alzheimer’s disease (Figure 4c). To further screen for hub genes, ML algorithms were employed. First, LASSO analysis identified four genes: *CASP8, GATM, LAP3*, and PPIF (Figures 4D,E). Next, the SVM-RFE algorithm selected 11 genes: *UCP2, CASP8, LAP3, GATM, PPIF, KMO, PABPC5, BID, CYP27A1, SLC25A19,* and *ME2* (Figures 4F,G). Simultaneously, the RF algorithm detected seven genes, namely, *GATM, CASP8, UCP2, ME2, BID, LAP3,* and *KMO*, that had a relative importance score above 1, (Figures 4H,I). Finally, the intersection of the genes selected by all three algorithms yielded three hub Mito-DEGs: *CASP8, GATM,* and *LAP3* (Figure 4J).

### 3.4 Analysis of gene co-expression and diagnostic accuracy in samples of AS

**FIGURE 5:**
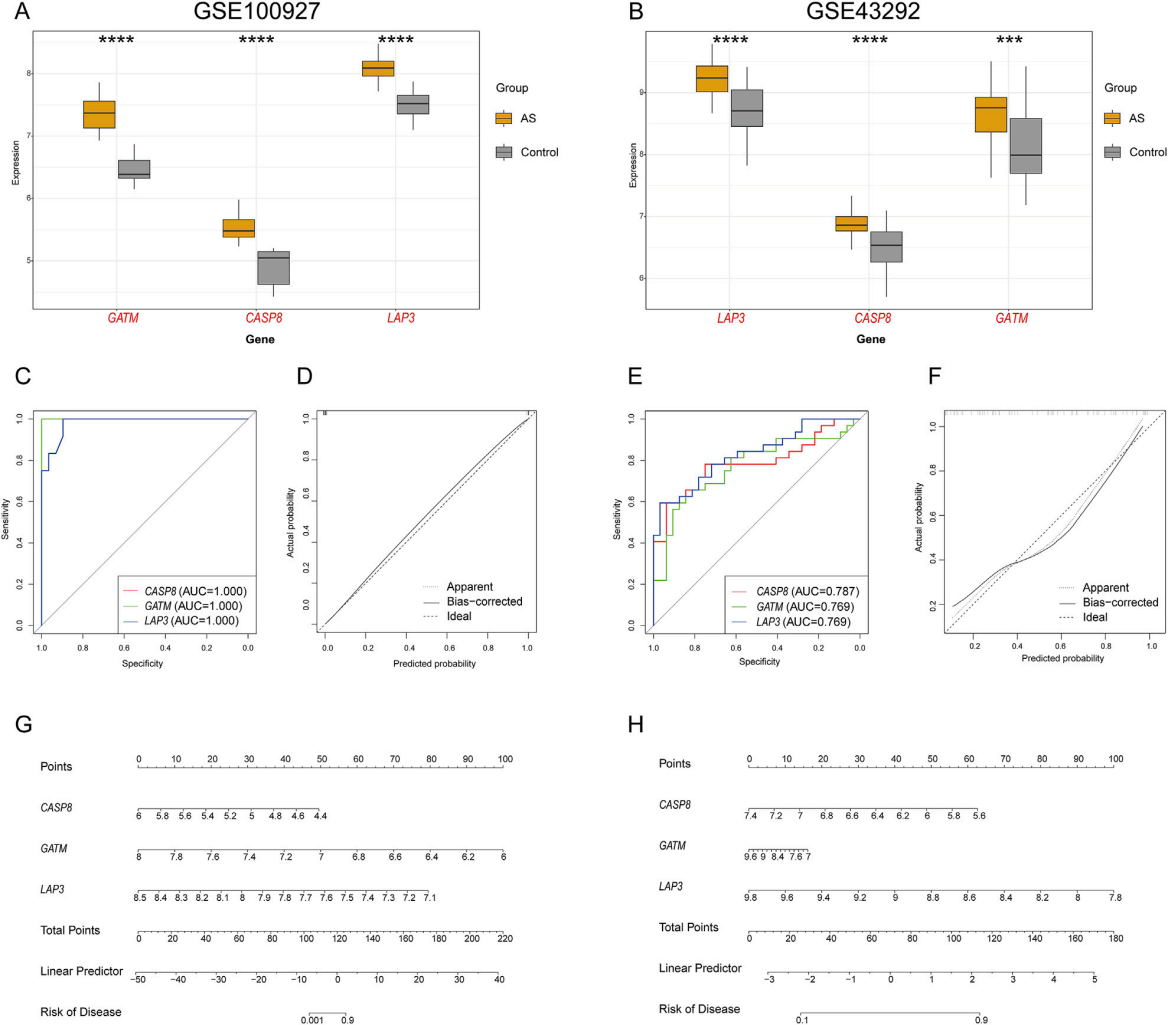
Analysis of the three feature genes. Expression levels of hub genes in **(A)** GSE100927 and **(B)** GSE43292. Upregulated genes in the AS group are highlighted in red. ROC curve analysis of hub genes in **(C)** GSE100927 and **(E)** GSE43292. Alignment diagram for predicting AS in **(G)** GSE100927 and **(H)** GSE43292. Calibration curve assessing the predictive accuracy of the model in **(D)** GSE100927 and **(F)** GSE43292.

Our analysis of the GSE100927 datasets noted a significant upregulation of three genes (*GATM, CASP8*, and *LAP3*) expression in the carotid AS group compared to the controls (Figure 5A). The diagnostic performance of the three hub genes in the GSE100927 dataset as biomarkers for AS was assessed using ROC curves (Figure 5C). Notably, the AUC values for *GATM* and *CASP8* were 1, and the AUC for *LAP3* was 0.981, demonstrating strong diagnostic potential for these three genes. A nomogram was then constructed based on these three feature genes, demonstrating their strong capability to predict the risk of AS (Figure 5G). The calibration curve (Figure 5D) showed that the nomogram had a high degree of accuracy in predicting the risk of AS. Similarly, upregulated expression of these genes in the AS group was validated in the GSE43292 datasets (Figure 5B), with the AUC for all gene datasets exceeding 0.7, further supporting their diagnostic performance (Figure 5E). The nomogram was used to further predict the risk of AS (Figure 5H), and the calibration curve confirmed the nomogram’s high predictive accuracy (Figure 5F).

### 3.5 Immune cell landscape in AS

**FIGURE 6:**
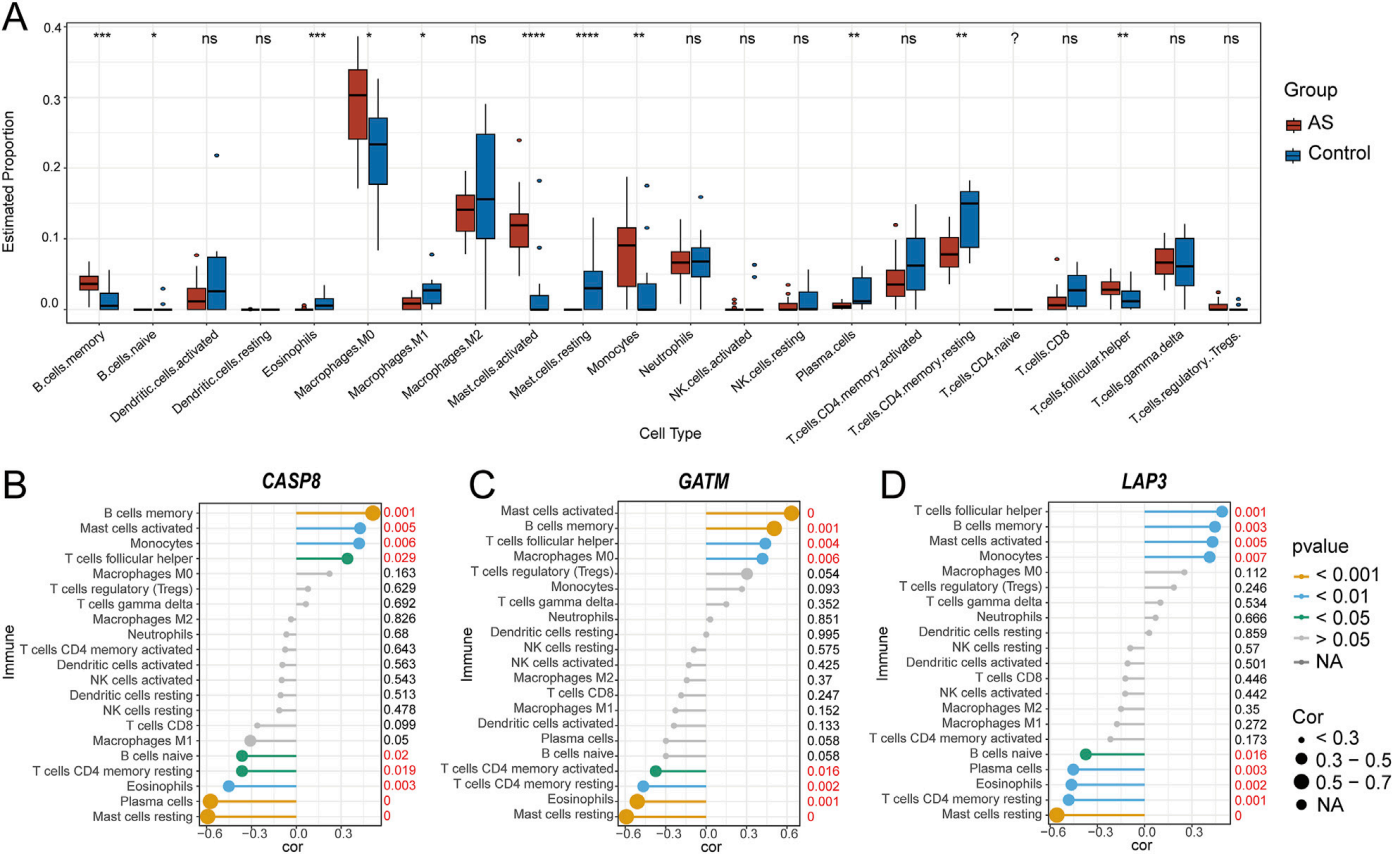
Visualization of immune cell infiltration distribution and the correlation between hub genes and infiltrating immune cells. **(A)** Comparison of 22 immune cell subtypes between carotid atherosclerosis samples and carotid artery control samples. Red represents AS samples, and blue represents normal samples. *P < 0.05, **P < 0.01, ***P < 0.001, ****P < 0.0001 (Wilcoxon–Mann–Whitney test); Correlation between *CASP8* **(B)**, *GATM* **(C)**, *LAP3* **(D)** and infiltrating immune cells. Gene expression values for biomarkers were obtained from the GSE100927 dataset for correlation analysis.

Evidence suggests that the development of atherosclerotic plaques is a persistent inflammatory process, involving both innate and adaptive immune systems (Wolf and Ley, 2019). To further elucidate the involvement of the immune system in the progression of AS, the LM22 signature matrix from the CIBERSORT algorithm, which contains characteristic gene expression profiles of 22 human immune cell subtypes, was utilized. Based on microarray data, supervised deconvolution analysis was performed to infer the relative proportions of different immune cells in plaque and control groups (Wang et al., 2023a). First, we examined the immune cell type abundance in carotid AS and control samples from the GSE100927 dataset (Supplementary Figure S5). Results indicated that the abundance of activated mast cells, monocytes, memory B cells, T follicular helper cells, and M0 macrophages was higher in the carotid AS samples (Figure 6A), whereas resting mast cells, plasma cells, CD4 memory resting T cells, eosinophils, M1 macrophages, and naive B cells were less abundant. Lastly, correlation analysis was executed between hub genes and immune cells. *CASP8* exhibited a positive correlation with memory B cells (r = 0.512, P < 0.001) and a negative correlation with resting mast cells (r = −0.593, P < 0.001) and plasma cells (r = −0.575, P < 0.001) (Figure 6B). *GATM* was positively correlated with activated mast cells (r = 0.634, P < 0.001) and memory B cells (r = 0.506, P < 0.001), and negatively correlated with resting mast cells (r = −0.592, P < 0.001) and eosinophils (r = −0.511, P < 0.001) (Figure 6C). *LAP3* showed a positive correlation with T follicular helper cells (r = 0.491, P < 0.001), memory B cells (r = 0.446, P < 0.001), and activated mast cells (r = 0.431, P < 0.001), while being negatively correlated with resting mast cells (r = −0.557, P < 0.001) (Figure 6D).

### 3.6 Single-cell data analysis

Previous analyses identified three hub Mito-DEGs: *CASP8*, *GATM*, and *LAP3*, which have been shown to have a positive relationship with mast cells, memory B cells, and follicular helper T cells through immune infiltration analysis. All three genes were also linked to monocyte-macrophages. Given the significant role of the inflammatory phenomenon, particularly monocyte-macrophages, in the development and progression of AS (Neupane et al., 2019), we proceeded to analyze the single-cell dataset from the GSE159677.

**FIGURE 7:**
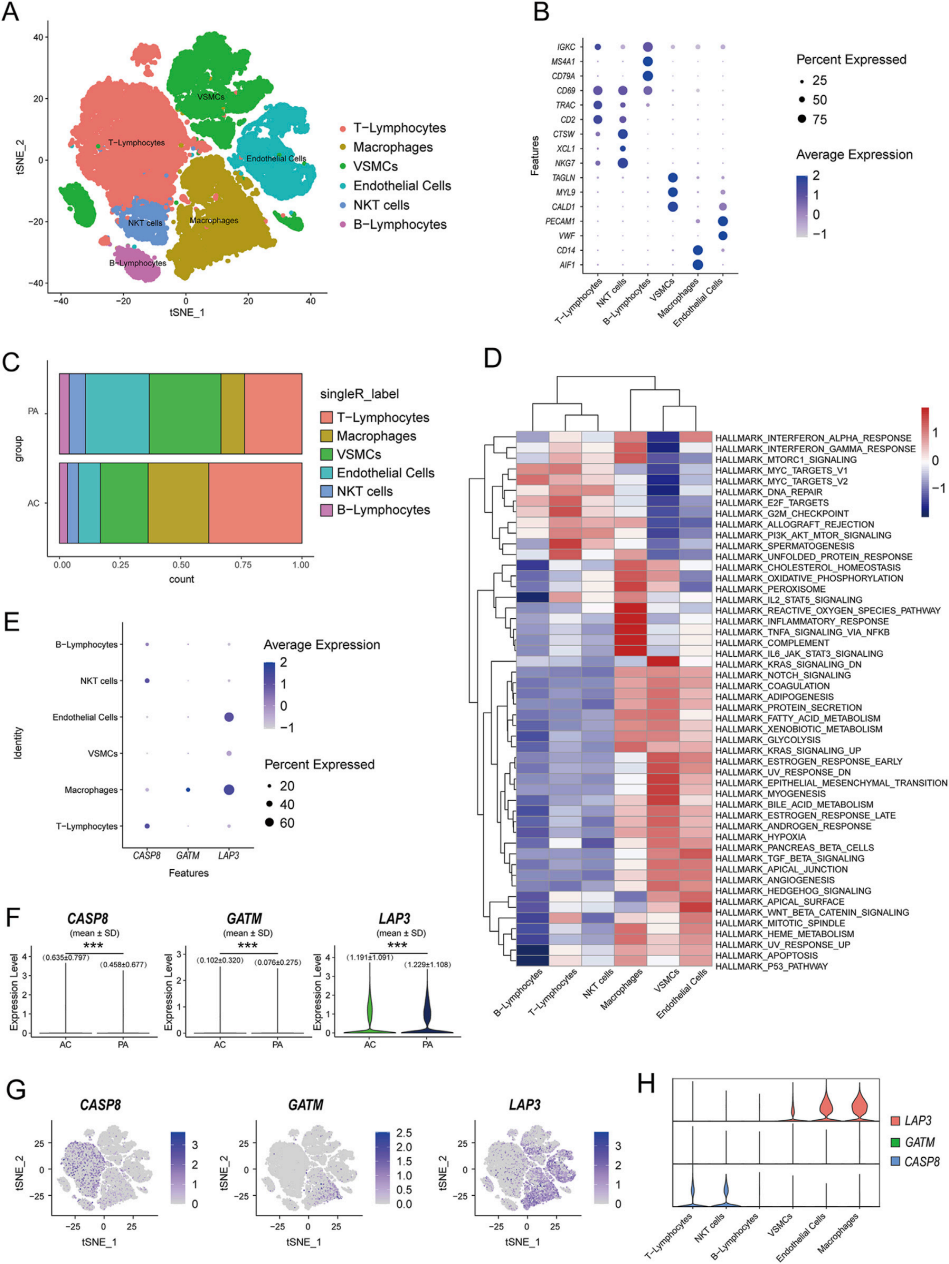
Identification of the expression and distribution of mitochondria-related hub genes by single-cell analysis. **(A)** UMAP dimension reduction analysis of major cell types in the GSE159677 dataset; **(B)** marker genes of 6 clusters of cells **(C)** Cluster distribution of cell types in the AC and PA groups; **(D)** Pathway enrichment analysis of the corresponding cell populations; **(E)** Dot plots showing the expression proportions of hub Mito-DEGs in the annotated 6 clusters of cells; **(F)** The expression profiles of the 3 hub Mito-DEGs between AC and PA **(G,H)** Violin-scatter plots and UMAP showing the distribution of the 3 hub Mito-DEGs across major immune cell types.

UMAP dimensional reduction analysis revealed the annotation of scRNA-seq data, identifying 6 major cell clusters: macrophages, endothelial cells (ECs), vascular smooth muscle cells (VSMCs), natural killer T (NKT) cells, T lymphocytes, and B lymphocytes. The differential gene expression between the six major cell types was evaluated using marker genes from previous studies (Wirka et al., 2019; Alsaigh et al., 2022) (Figures 7A,B). The same cell types were identified in both the atherosclerotic core (AC) group and the patient-matched proximal adjacent (PA) group Supplementary Table S1, but the proportions of each cell type differed markedly, with the PA group exhibiting a higher proportion of endothelial cells. This may be attributed to the ability of macrophages to promote tissue repair and proliferation of vascular smooth muscle cells during disease progression, thereby increasing plaque stability. However, advanced atherosclerotic plaques are characterized by a high abundance of pro-inflammatory macrophages, which secrete matrix-degrading enzymes, induce cell death in surrounding tissues, and lead to plaque instability and rupture (De Meyer et al., 2024). Consistent with this, the single-cell transcriptome of the AC group showed higher numbers of T cells and macrophages, although the phenotypes of these cells may vary depending on the stage and location of the disease (Figure 7C). To further investigate the regulatory network variations in coronary artery plaques, we used the Hallmark gene sets to evaluate pathway differences between the corresponding cell populations. The analysis revealed that multiple pathways, including reactive oxygen species pathway, inflammatory response, TNF-α/NF-κB pathway, complement activation, and IL-6 signaling, were upregulated in macrophages (Figure 7D). We subsequently observed significant expression proportions of *GATM* and *LAP3* in macrophages, while *CASP8* showed significant expression in NK cells and T cells in the dot plots (Figure 7E). Quantitative analysis further revealed that the expression levels of all three DEGs differed significantly between the AC and PA groups (Figure 7F). This was additionally confirmed by UMAP and violin-scatter plots, which illustrated the level of expression and dispersion of these genes among immune clusters (Figures 7G,H). Studies have demonstrated that LDL and other factors infiltrating the arterial wall stimulate endothelial cells to produce pro-inflammatory molecules, including E-selectin, P-selectin, intercellular adhesion molecule-1, and vascular cell adhesion molecule-1. This facilitates the attachment and migration of immune cells, including monocytes, T cells, B cells, NK cells, and also dendritic cells (DCs), to the vessel wall (Thorp et al., 2011; Tacke et al., 2007; Libby et al., 2008). After macrophages engulf lipids and form foam cells, they, along with recruited T cells, accumulate in the arterial wall, therefore playing a role in the inflammatory response and progression of plaque (Gotsman et al., 2008). DCs are directly involved in cholesterol homeostasis and immune responses, and thus offer a new avenue for research into atherosclerotic plaques (Gautier et al., 2009; Paulson et al., 2010).

### 3.7 Cell-cell interaction analysis and pseudotime trajectory

**FIGURE 8:**
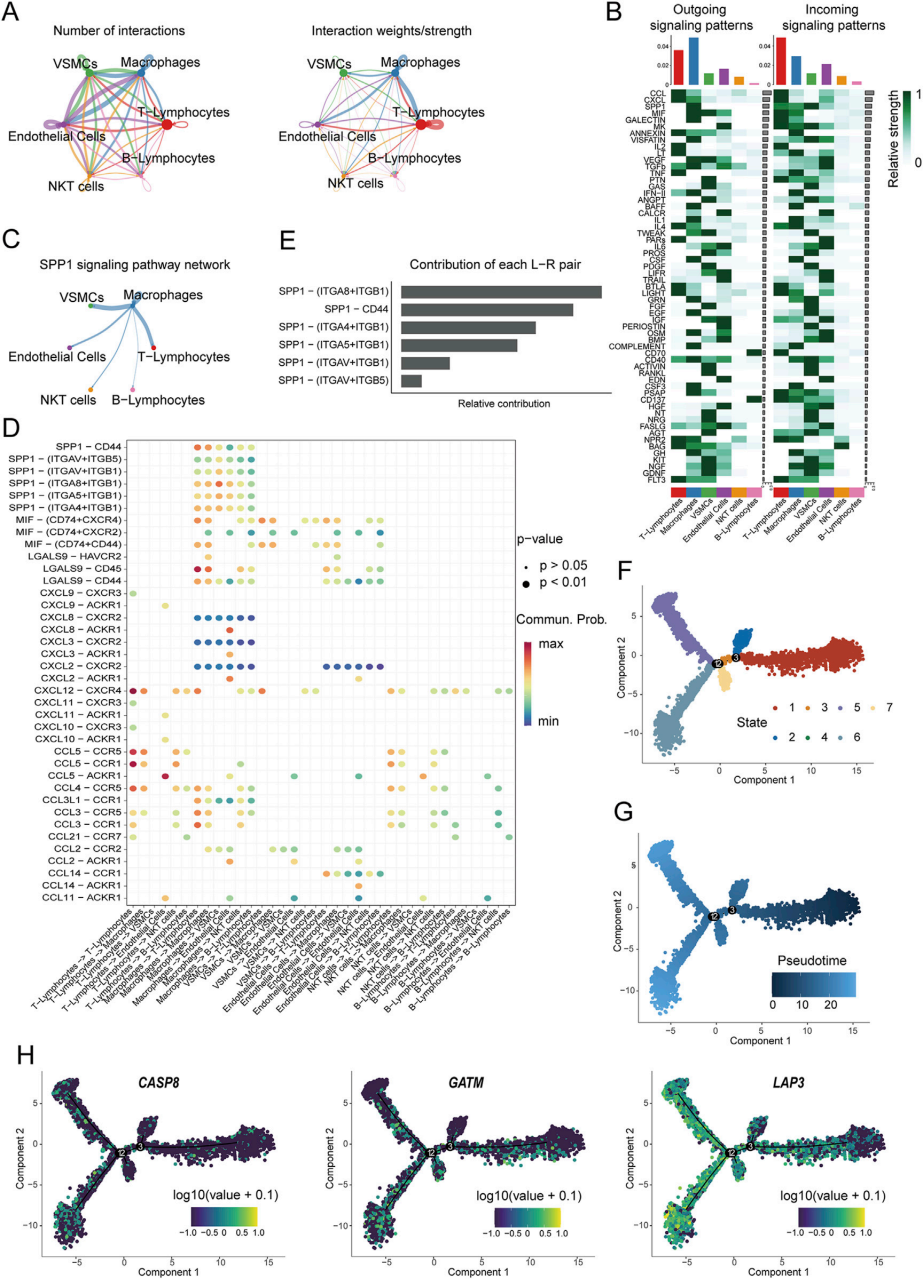
Cell communication analysis and developmental trajectory. **(A)** Communication hubs and weights among various cell types in the annotated 6 clusters of cells; **(B)** Relative intensity of incoming and outgoing signaling patterns in different cell clusters; **(C)** Inferred SPP1 signaling pathway network; **(D)** Main communication pathways between macrophages and other cell types; **(E)** Relative contribution of receptor-ligand pairs; **(F)** UMAP plot of different states of macrophages; **(G)** Pseudotime analysis of macrophages; **(H)** Predicted expression patterns of Mito-DEGs during the pseudotime process.

The single-cell analysis of carotid artery plaques identified 6 major cell clusters, among which T cells, endothelial cells, and macrophages demonstrated enriched intercellular communication (Figure 8A). A total of 59 signaling pathways were detected between these cell clusters, with T cells and macrophages exhibiting the most signaling pathways. Notably, the SPP1 signaling pathway displayed a prominent signaling pattern in macrophages (Figure 8B). In addition, the macrophage cell cluster exhibited high expression within the SPP1 signaling pathway (Figure 8C). To further investigate the biological functions of macrophages, receptor-ligand analysis was performed. The contribution of each ligand-receptor pair to the signaling pathway was visualized, revealing that SPP1 − (ITGA8+ITGB1), SPP1 − CD44, and SPP1 − (ITGA4+ITGB1) made significant contributions (Figures 8D,E). Subsequently, pseudotime trajectory analysis was performed to infer the pathways of macrophage state transitions and developmental trajectories within the AS clusters (Figures 8F,G). Cellular developmental relationships were reconstructed, revealing an orderly progression of cell states over pseudotime. For the selected Mito-DEGs, *LAP3* exhibited fluctuations over time, indicating dynamic changes during the developmental process, while *CASP8* and *GATM* remained relatively stable throughout development (Figure 8H).

### 3.8 *In vitro* analyses

**FIGURE 9:**
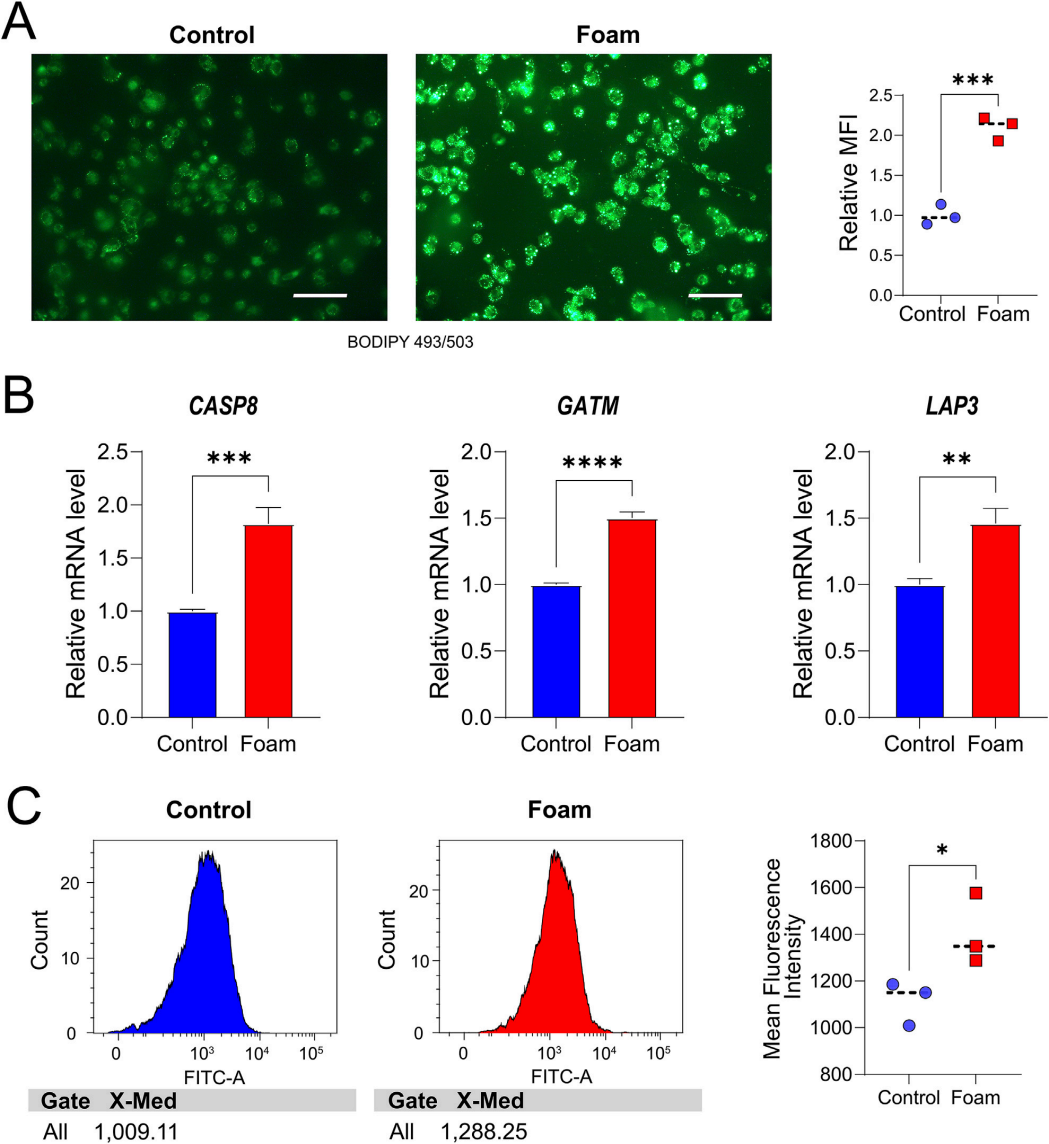
*In vitro* Analyses. **(A)** BODIPY 493/503 staining of THP-1 cells in the control group. Scale bar, 100 μm, and the foam cell group **(B)** Validation of hub Mito-DEG expression, with relative mRNA levels of *CASP8*, *GATM*, and *LAP3* in THP-1 cells. **P < 0.01, ***P < 0.001, ****P < 0.0001. **(C)** The mean fluorescence intensity (MFI) of mitochondria in the two groups was analyzed by flow cytometry using Mito-Tracker Green-labeled cells.

To validate the role of hub genes in foam cell formation (a key pathological feature of AS, given that the lipid core of AS primarily consists of foam cells), an *in vitro* model was developed. In this study, THP-1 cells were stimulated with 50 μg/mL ox-LDL for 48 h, followed by staining with the green fluorescent probe BODIPY 493/503 to visualize intracellular lipid droplets, thereby confirming successful foam cell formation. Results showed a significant increase in BODIPY 493/503 staining in ox-LDL-treated THP-1 cells compared to the control group, indicating a greater accumulation of intracellular lipid droplets and the formation of foam cells (Figure 9A). In comparison to the control (Supplementary Table S2), the foam cell group exhibited significantly elevated mRNA expression of *CASP8* (P = 0.0008), *GATM* (P < 0.0001), and *LAP3* (P = 0.0029) (Figure 9B), consistent with the earlier analysis. Finally, flow cytometry was used to assess mitochondrial mean fluorescence intensity (MFI), revealing increased mitochondrial abundance/mass in the foam group (*P* = 0.048) (Figure 9C).

## 4 Discussion

Mitochondria are essential subcellular organelles in mammalian cells. Perturbations in mitochondrial homeostasis and dynamics can induce reactive oxygen species (ROS), accelerating cellular senescence (Madamanchi and Runge, 2007; Ježek et al., 2018; You et al., 2023). In our *in vitro* experiments, THP-1 cells stimulated with ox-LDL exhibited increased mitochondrial fluorescence signals compared to controls, suggesting mitochondrial biogenesis or fission events that expedite foam cell formation. Natarajan et al. proposed that chronic inflammatory stimuli in AS enhance mtDNA synthesis, promoting mitochondrial biogenesis and thereby exacerbating AS (Natarajan et al., 2024). Moreover, in hypoxic rats, Drp1-mediated mitochondrial fission induces myocardial aging (You et al., 2023). Thus, mitochondria play a pivotal role in the pathogenesis of the disease. AS is one of the major diseases that threaten human health. Typically, the subclinical stage of AS persists without being noticed until a significant cardiovascular event occurs in an individual (Singh et al., 2018; Merida et al., 2024). Carotid intima-media thickness (Yang et al., 2020) and the presence of carotid plaques (Sillesen et al., 2012) are currently known early markers of AS. Therefore, selecting an appropriate disease model is crucial for identifying biomarkers in the subclinical stage of AS. In this study, carotid plaque samples and controls from the GSE100927 dataset were analyzed, along with 64 carotid artery samples with or without AS plaques from the GSE43292 dataset, so as to identify mitochondria-related hub genes involved in the progression of AS.

The present work successfully identified 815 genes with similar expression trends across both datasets. KEGG pathway enrichment analysis of the DEGs highlighted remarkable enrichment in the PI3K−Akt signaling pathway, lipid and atherosclerosis, as well as immune-related processes. After intersecting these DEGs with the most significant module genes identified through WGCNA, twelve mitochondria-related genes were identified (*PPIF, SLC25A19, ME2, GATM, LAP3, CYP27A1, KMO, UCP2, BID, C15orf48, CASP8, and PABPC5*). GO and KEGG enrichment analyses indicated that these genes were remarkably enriched in pathways related to apoptosis, arginine and proline metabolism, p53 signaling pathway, regulation of mitochondrial membrane potential, mitochondrial inner membrane, death receptor binding, and oxidoreductase activity. The findings suggested that abnormalities in lipids and immunity, apoptosis, impaired mitochondrial membrane function, and oxidoreductase activity may play crucial roles in the disease process. Mitochondria, as regulators of key processes including ATP production, ROS homeostasis, and apoptosis, play a central role in determining cell fate, with mitochondrial-encoded genes accounting for less than 1% of the genome (Fontana and Gahlon, 2020). Functional abnormalities in this small subset of mitochondrial genes can amplify pathological effects. Studies have shown that the NLRP3 inflammasome senses mitochondrial dysfunction, thereby amplifying inflammatory responses and triggering a cascade of downstream effects (Zhou et al., 2011). In atherosclerotic tissues, the prevalence of certain individual mitochondrial mutations is relatively high (Sobenin et al., 2012). These findings suggest that although mitochondrial genes represent a numerically minor fraction and may not appear advantageous in terms of disease progression at first glance, their critical regulatory functions and cascade effects may accelerate disease development. Accordingly, further analysis of these 12 mitochondria-associated genes was conducted.

Three mitochondria-related hub genes, *CASP8*, *GATM*, and *LAP3*, were identified using 3 ML algorithms. Caspase-8 (*CASP8*) encodes a protein in the cysteine-aspartic acid protease (caspase) family. This enzyme is involved in initiating the intrinsic apoptotic pathway when cells are exposed to DNA-damaging stressors such as UV or γ-radiation, cytotoxic agents, or cytokine deprivation, either from internal or external sources (Mandal et al., 2020). Upon activation, *CASP8* promotes the activation of the BH3 interacting domain death agonist, which induces mitochondrial outer membrane permeabilization. This process can lead to cytochrome c release and subsequently trigger the apoptotic cascade (Hung et al., 2021; Moldoveanu, 2023). Cytochrome c release and the subsequent activation of caspase-3 have been shown to occur in cardiomyocytes, in cases of human cardiomyopathy, during hypoxia-induced apoptosis in adult rat ventricular myocytes, in the induction of monocyte inflammation, and in the development of AS, contributing to peripheral arterial occlusive disease (PAOD) (Narula et al., 1999; de Moissac et al., 2000; Chen Y. C. et al., 2020; Zhang et al., 2021). Recent studies have shown that caspase-8 deficiency in macrophages exposed to oxidized low-density lipoprotein (ox-LDL) leads to increased MLKL phosphorylation and reduced apoptotic signaling. However, in caspase-8-deficient (*Casp8* ^*komac*^) mice, lack of MLKL phosphorylation results in increased susceptibility to necroptosis within atherosclerotic plaques. These findings indicate that caspase-8 exerts a dual regulatory effect, facilitating apoptosis while suppressing necroptosis, which actively contributes to plaque progression and instability in AS (Pilot et al., 2025). These findings underscore the significance of the CASP8-mediated apoptotic cascade in CVD.

The glycine amidinotransferase gene (*GATM*, chromosome 15q15.3) is also known as L-Arginine:amidinotransferase (AGAT, EC 2.1.4.1). Its primary function is to facilitate the initial crucial stage of endogenous creatine biosynthesis by transforming L-Arginine (Arg) and glycine into ornithine and guanidinoacetate (GAA) (Humm et al., 1997; Tsikas and Wu, 2015; Khan et al., 2016; Haghikia et al., 2017; Baker et al., 2021). Creatine is an organic compound that is reversibly phosphorylated by creatine kinase (CK), functioning as a buffer to maintain intracellular ATP levels (Joncquel-Chevalier Curt et al., 2015), which is why most research on *GATM* has focused on its role in kidney diseases and cerebral creatine deficiency syndromes (CCDS) (Reichold et al., 2018; Goldstein et al., 2024; He et al., 2024). Arg serves as a substrate in the biosynthesis of nitric oxide (NO), a potent endogenous vasodilator (Moncada and Higgs, 1993; Leiper and Vallance, 1999). Increased GATM expression and creatine synthesis have been reported in the myocardium of patients with heart failure (Cullen et al., 2006). Additionally, certain expression quantitative trait loci (eQTL) of *GATM* have been reported to be significantly associated with statin-induced myopathy (Mangravite et al., 2013; Norata et al., 2014), which might offer new insights into the considerable interindividual variability in the response to statins for lowering plasma LDL concentrations in cardiovascular disease patients (Simon et al., 2006). Studies have found that *GATM* expression is enhanced in polarized M2 macrophages, indicating its potential involvement in the etiology of associated inflammatory disorders (Yu et al., 2022).

Leucine aminopeptidase 3 (*LAP3*), a key member of the LAP family, is closely linked to tumor cell proliferation, migration, and malignancy grade (He et al., 2015; Fang et al., 2019; Kuhara et al., 2021). LAP3 is remarkably upregulated in hyperinflammation-related diseases and is deemed a potential target for anti-inflammatory drugs (Didangelos, 2020). An *in vivo* study indicated an upregulated *LAP3* expression in the hepatocytes and serum of rats fed a high-fat diet, which induces non-alcoholic fatty liver disease (NAFLD) and inhibits autophagy in LO2 cells (Feng L. et al., 2022). These findings denoted that *LAP3* may be important in inflammation and lipid accumulation, providing a promising avenue for further exploration.

Inflammatory responses are involved throughout the entire progression of AS. In the GSE100927 dataset, CIBERSORT was applied to assess the immune cell infiltration in the peripheral blood of individuals with coronary artery disease. In AS samples, the abundance of activated mast cells, monocytes, memory B cells, T follicular helper cells, and M0 macrophages was higher. This aligns with previous findings (Xiong et al., 2022; Wang F. et al., 2024), where inflammatory and immune cells (macrophages, T cell subsets, and mast cells) coordinate the development of intimal atherosclerotic lesions (Poto et al., 2024). Recent research (Xiang et al., 2023) has shown that in patients with atherosclerosis, increased expression of Morrbid in monocytes and arterial walls promotes the differentiation of monocytes into M0 macrophages, suggesting enhanced recruitment of M0 macrophages to plaque sites in the AS microenvironment. From a functional perspective, M0 macrophages can undergo polarization into different subtypes with distinct roles. Mast cells are involved in promoting neutrophil recruitment and the formation of extracellular traps, exacerbating the inflammatory response, which can lead to plaque rupture and thrombosis (Elieh-Ali-Komi et al., 2024). Additionally, a study by Kritikou et al. demonstrated that adoptive transfer of CD1d^−/−^ or control mast cells into mast cell-deficient apoE^−/−KitW-sh/W−sh^ mice, followed by an atherogenic diet, resulted in larger atherosclerotic plaques and increased secretion of inflammatory factors in reconstituted apoE^−/−KitW-sh/W−sh^ mice, indicating that mast cells exacerbate the progression of atherosclerosis through their pro-inflammatory activity (Kritikou et al., 2019). T follicular helper cells activate B2 cells, which could express IgG antibodies, and elevated IgG levels have been confirmed to be linked to the progression of AS (Wolf and Ley, 2019; Wang F. et al., 2024). Maintaining the balance of various macrophage phenotypes is crucial in AS formation and progression, as it determines the outcome of the inflammatory response (Mantovani et al., 2009). M0 macrophages play a key role in maintaining this balance and can be polarized into either the pro-inflammatory M1 subtype or the anti-inflammatory M2 subtype, depending on cytokines and chemokines present in the microenvironment (Adamson and Leitinger, 2011). Prior research has shown that a key feature of AS regression is the overall reduction of plaque macrophages and the enrichment of selectively activated M2 macrophage markers (Rahman et al., 2017). In this study, among all differentially enriched immune cells, the abundance of M0 macrophages were negatively correlated with M2 macrophages, which may indicate persistent inflammation during the disease’s progression. This aligns with findings in advanced AS lesions, showing that M2 macrophages were less abundant and that the relative abundance of macrophage phenotypes for M1, Mox, and M2 was approximately 40%, 30%, and 20%, respectively (Kadl et al., 2010). The hub Mito-DEGs identified in this study exhibited a strong positive correlation with memory B cells, activated mast cells, and T follicular helper cells. Advances in single-cell RNA sequencing (scRNA-seq) technology have provided unprecedented opportunities for identifying cellular populations and their markers in various diseases (Sun et al., 2022). Further single-cell level analysis confirmed that two of the hub Mito-DEGs, *GATM* and *LAP3*, were enriched in macrophages, while *CASP8* showed increased expression in NK cells and T cells. Additionally, cell-cell communication analysis and developmental trajectory exploration identified relevant regulatory mechanisms. In subsequent *in vitro* analysis, we used THP-1 cells to construct an atherosclerotic plaque model, where foam cells exhibited higher expression of *CASP8*, *GATM*, and *LAP3*. These results are consistent with our earlier analyses, further supporting the role of these hub Mito-DEGs in AS.

### 4.1 Future perspectives for low- and middle-income countries (LMICs)

In the past, atherosclerosis was regarded as an inevitable, progressive, and degenerative condition associated with aging, primarily affecting developed countries. However, improvements in sanitation, widespread vaccination, and effective treatment of acute infections have reduced the burden of communicable diseases in developing countries, resulting in increased survival and a corresponding rise in chronic conditions such as atherosclerosis (Libby, 2021). Concurrently, the westernization of lifestyles has led to a marked increase in the burden of atherosclerosis among women, young adults, individuals from diverse ethnic backgrounds, and older populations in LMICs (Fowkes et al., 2017; Libby, 2021; Nedkoff et al., 2023). Unlike the age-associated form once deemed unavoidable, this type of atherosclerosis is preventable and modifiable through lifestyle changes and medical interventions. The application of polygenic risk scores can help identify young individuals who may derive particular benefit from early preventive strategies. The mitochondrial-immune regulatory network identified in this study provides a novel approach for low-cost screening and intervention in LMICs. With continued validation of AS-related mechanisms and refinement of clinical diagnostics, these findings are expected to offer substantial benefits to LMICs.

Strengths and limitations: The present work is the first that we are aware of that integrates mitochondrial-related genes with AS using bioinformatics to identify hub Mito-DEGs, and to validate the expression of hub genes at the *in vitro* cellular level. However, there are certain limitations. Firstly, since the carotid artery is a superficial vessel and often the first choice for early AS detection, our study focused on datasets from carotid AS and control groups. The identified related genes were able to distinguish between the disease group and the normal group; however, comparative testing with other diseases was lacking, resulting in limitations regarding the definitive diagnosis of AS. Therefore, blood, body fluids, and other samples will subsequently be collected from clinical AS patients for further validation. However, due to the limited availability of databases, future studies should aim to validate these findings using larger expression cohorts. Finally, while we identified hub Mito-DEGs, further validation is required to explore the detailed mechanisms of these genes in AS.

## 5 Conclusion

This study, through integrated bioinformatics analysis, identified three mitochondrial-related hub genes in AS: *CASP8*, *GATM*, and *LAP3*. *CASP8* promotes plaque instability by modulating the balance between macrophage apoptosis and necroptosis; *GATM* is involved in arginine metabolism and M2 macrophage polarization; *LAP3* contributes to AS progression by regulating inflammation and lipid accumulation. Immunoinfiltration analysis demonstrated significant enrichment of M0 macrophages, activated mast cells, and T follicular helper cells in AS plaques, all positively correlated with hub gene expression. *In vitro* experiments confirmed that the expression levels of all three genes were significantly elevated in foam cells. This study is the first to identify a mitochondrial-immune regulatory network, offering novel targets for the identification of early biomarkers in AS.

## Data Availability

The original contributions presented in the study are included in the article/Supplementary Material, further inquiries can be directed to the corresponding author.

## Supplementary material

SUPPLEMENTARY FIGURE 1 Hierarchical clustering illustrates sample correlations.

SUPPLEMENTARY FIGURE 2 Results of DEG and GO-KEGG Analysis in Upregulated Genes. **(A, C)** Enriched GO terms for upregulated genes in GSE100927 and GSE43292; **(B, D)** KEGG pathway enrichment results for upregulated genes in GSE100927 and GSE43292.

SUPPLEMENTARY FIGURE 3 Results of DEG and GO-KEGG Analysis in Downregulated Genes. **(A, C)** Enriched GO terms for downregulated genes in GSE100927 and GSE43292; **(B, D)** KEGG pathway enrichment results for downregulated genes in GSE100927 and GSE43292.

SUPPLEMENTARY FIGURE 4 Visualization of the scale-free fit index (left) and mean connectivity (right) for different soft threshold powers (β). The red line indicates a correlation coefficient of 0.85.

SUPPLEMENTARY FIGURE 5 Percentage stacked bar chart showing the relative composition of immune cell proportions in each sample.

## Abbreviations

AS, Atherosclerosis; ASCVD, Atherosclerotic cardiovascular disease; BP, biological process; CC, cellular component; CCDS, cerebral creatine deficiency syndromes; CVD, cardiovascular disease; DEGs, differentially expressed genes; FBS, fetal bovine serum; FC, fold change; GEO, Gene Expression Omnibus; IHD, ischaemic heart disease; LAP3, Leucine aminopeptidase 3; LASSO, least absolute shrinkage and selection operator; LDL, low-density lipoprotein; MF, molecular function; ML, machine learning; NAFLD, non-alcoholic fatty liver disease; ox-LDL, oxidized low-density lipoprotein; RF, random forest; ROC, receiver operator characteristic; TOM, topological overlap matrix; WGCNA, weighted gene co-expression network analysis.
