# Supplementary material for: The role of mitochondria-related genes and immune infiltration in carotid atherosclerosis: identification of hub targets through bioinformatics and machine learning approaches
Source: Front Genet. 2025 Aug 5;16:1597445. doi: 10.3389/fgene.2025.1597445 (PMC12361237; doi:10.3389/fgene.2025.1597445)
Supplement: Supplementary file 1 [file Table1.docx]

**Supplementary Table 1**. Basic patient information and carotid plaque classification in the GSE159677

| Patient | Age | Smoker | Diabetes (A1C) | Hypertension | Dyslipidemia | Statin | Symptomatic | AHA Classificati |
| --- | --- | --- | --- | --- | --- | --- | --- | --- |
| 1 | 82 | Yes | Yes (9.3%) | Yes | Yes | Yes | No | Type VII Calcified |
| 2 | 87 | No | Yes  (6.4%) | Yes | Yes | Yes | No | Type VII Calcified |
| 3 | 65 | Yes | No (5.5%) | Yes | Yes | Yes | No | Type VII  Calcified |

**Supplementary Table 2**. Ct values of housekeeping gene(GAPDH)

| *GADPH* Ct values  Group | | Replicate 1 | Replicate 2 | Replicate 3 |
| --- | --- | --- | --- | --- |
| Experiment 1 | Control | 12.52 | 12.67 | 12.55 |
|  | Form cell | 17.74 | 18.29 | 19.02 |
| Experiment 2 | Control | 16.33 | 16.36 | 16.33 |
|  | Form cell | 17.92 | 18.06 | 17.98 |
| Experiment 3 | Control | 12.52 | 11.3 | 13.56 |
|  | Form cell | 13.64 | 14.14 | 12.74 |
