# Supplementary figures and images for: The role of mitochondria-related genes and immune infiltration in carotid atherosclerosis: identification of hub targets through bioinformatics and machine learning approaches
Source: Front Genet. 2025 Aug 5;16:1597445. doi: 10.3389/fgene.2025.1597445 (PMC12361237; doi:10.3389/fgene.2025.1597445)

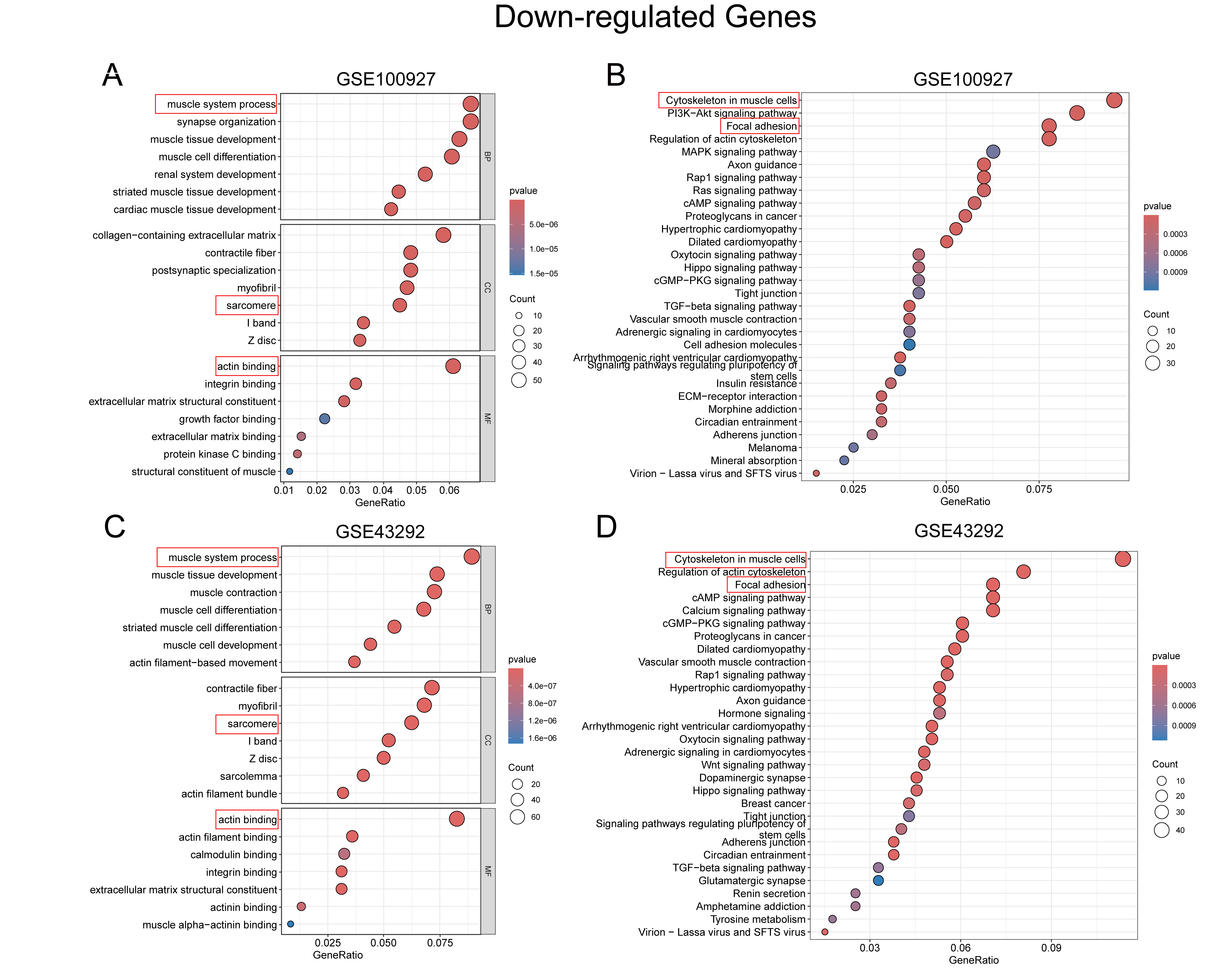

Supplement: Supplementary file 2 [file Image3.tif]

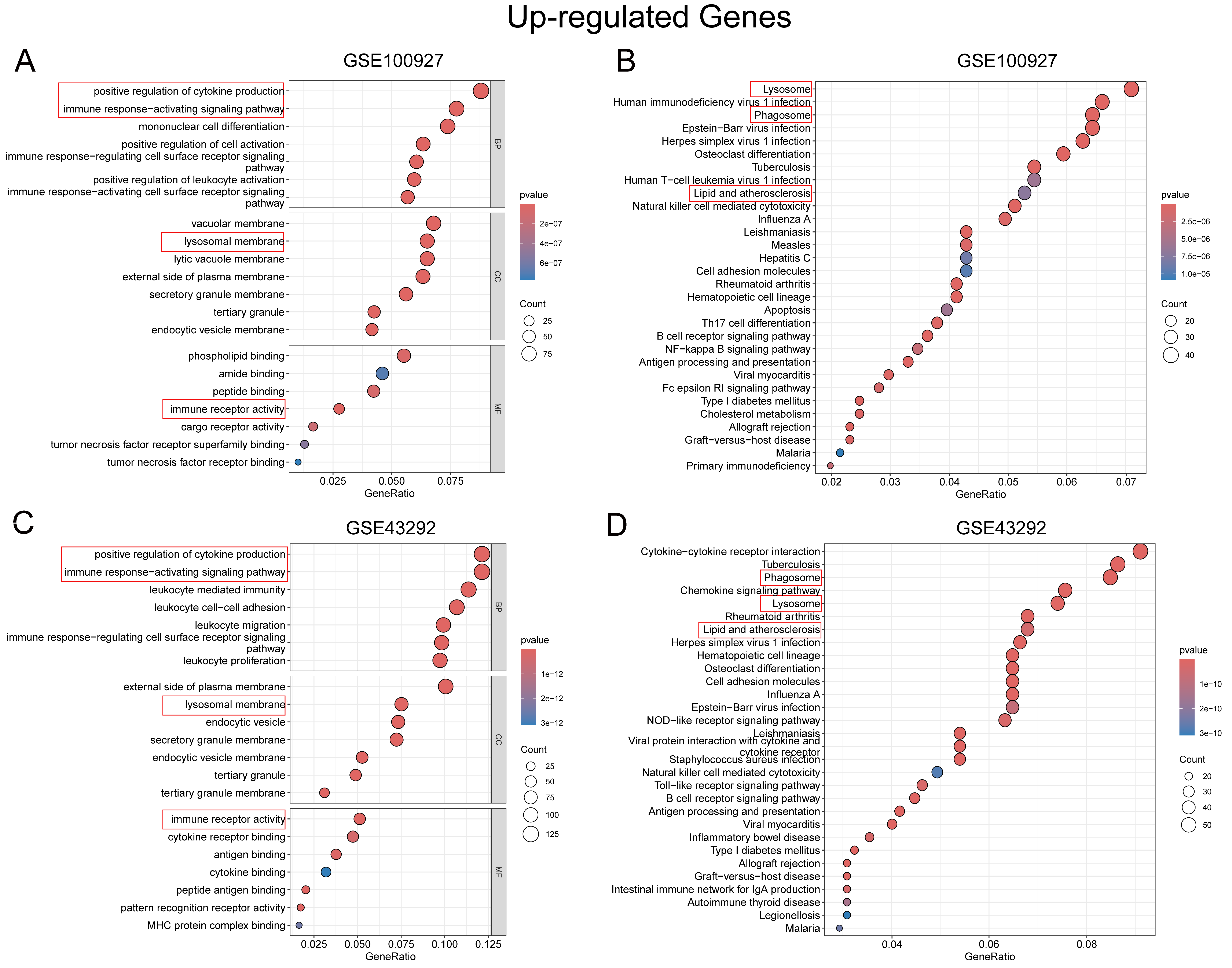

Supplement: Supplementary file 4 [file Image2.tif]

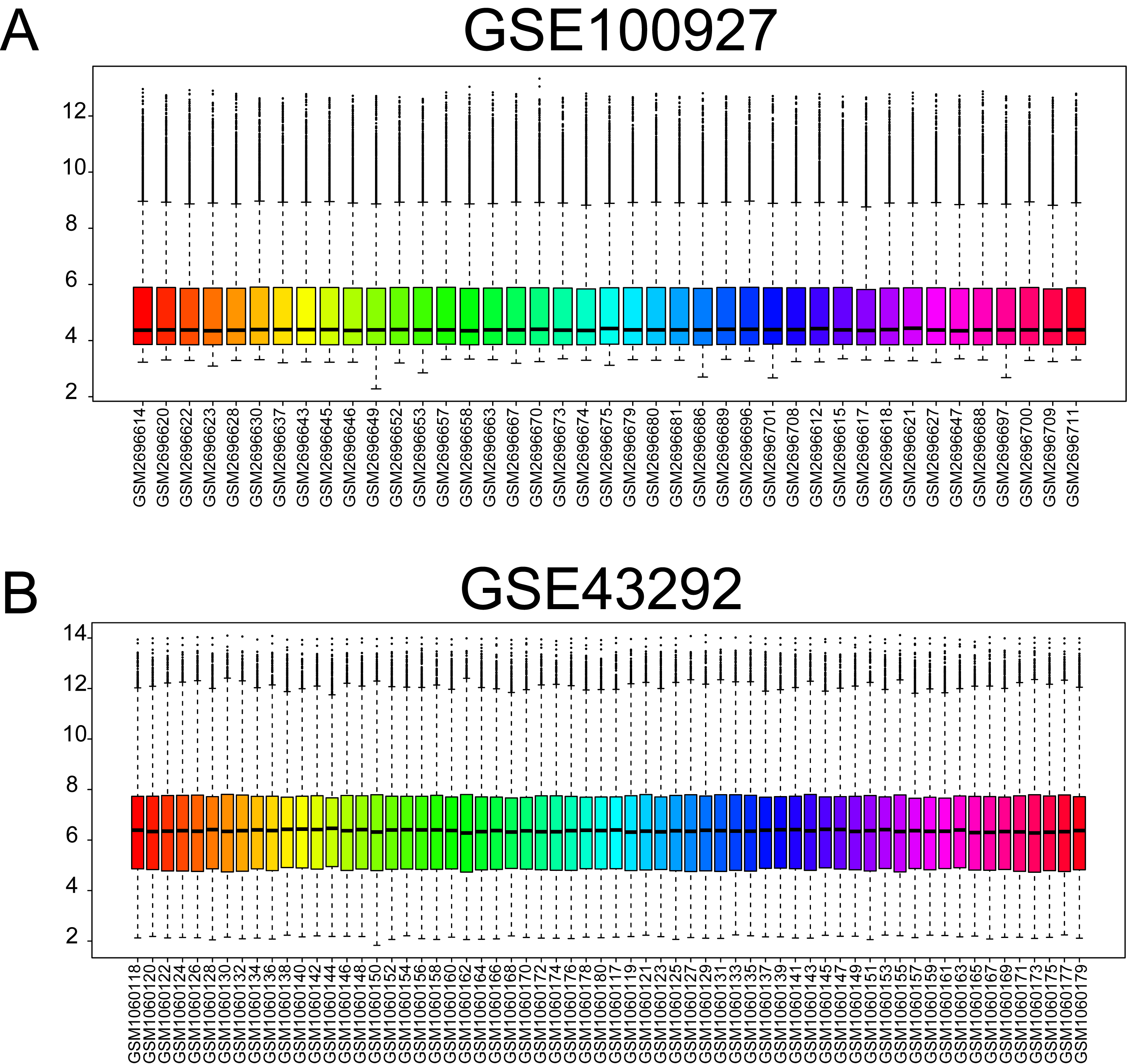

Supplement: Supplementary file 5 [file Image1.tif]

Scale independence

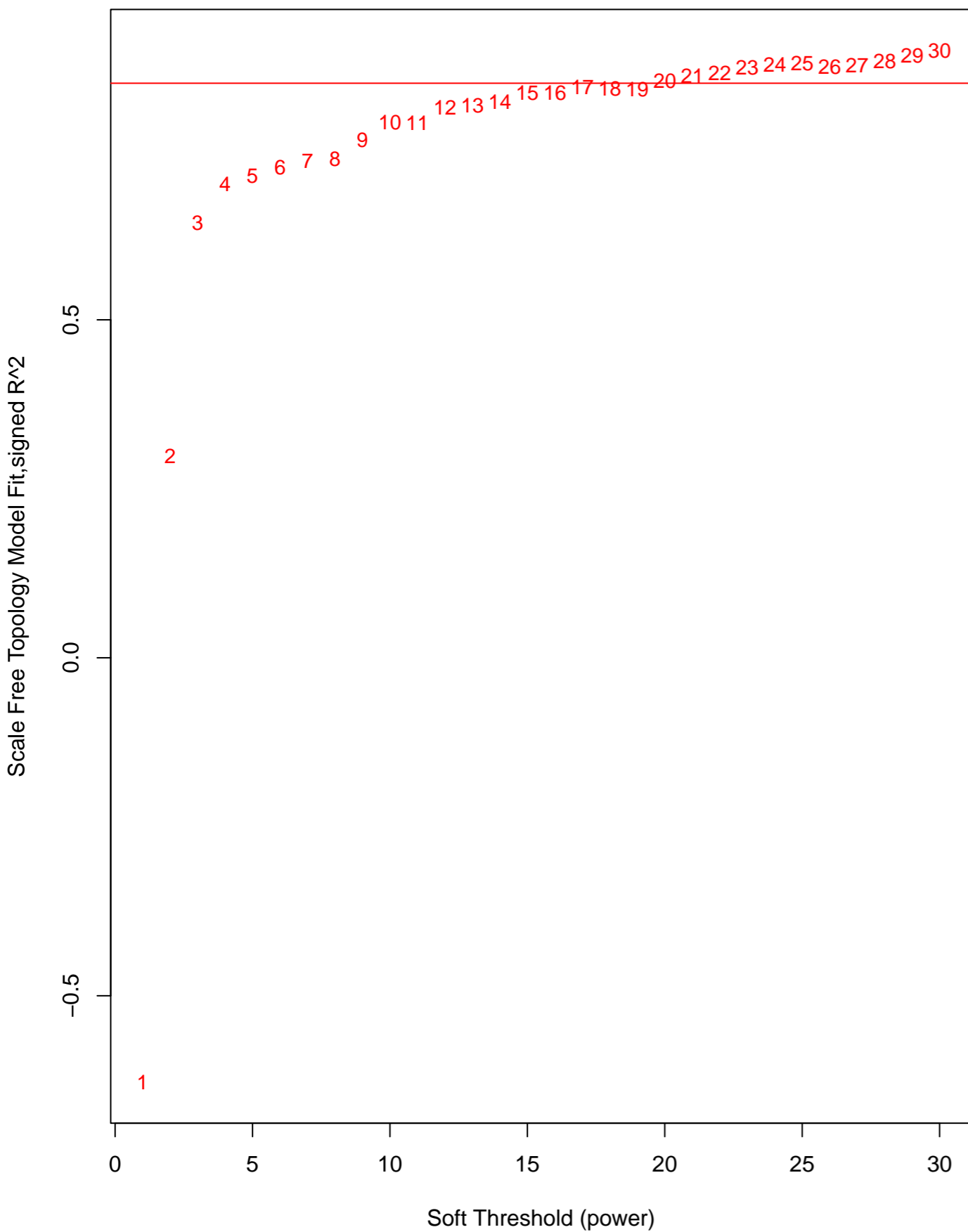

Mean connectivity

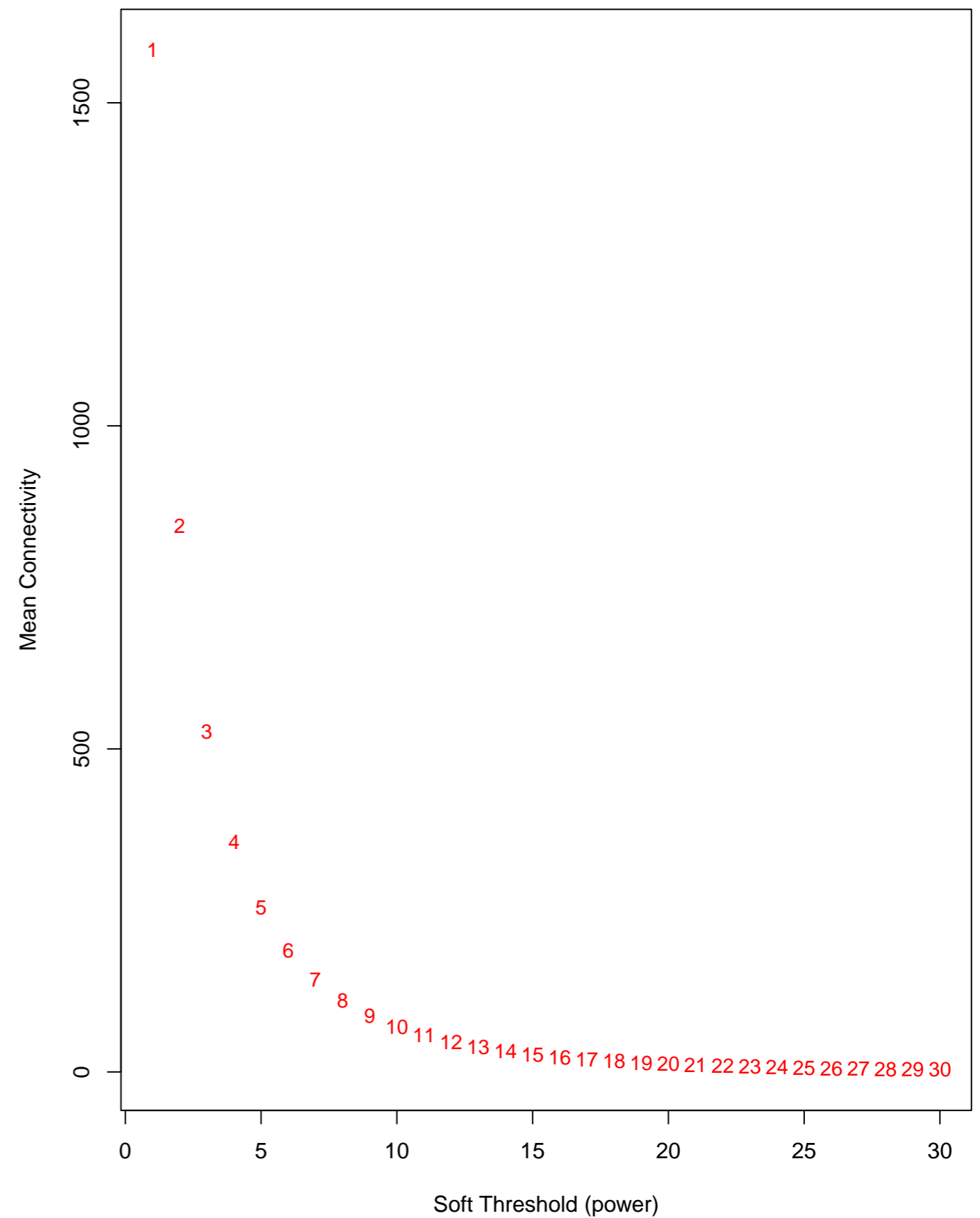

Supplement: Supplementary file 6 [file Image4.pdf]

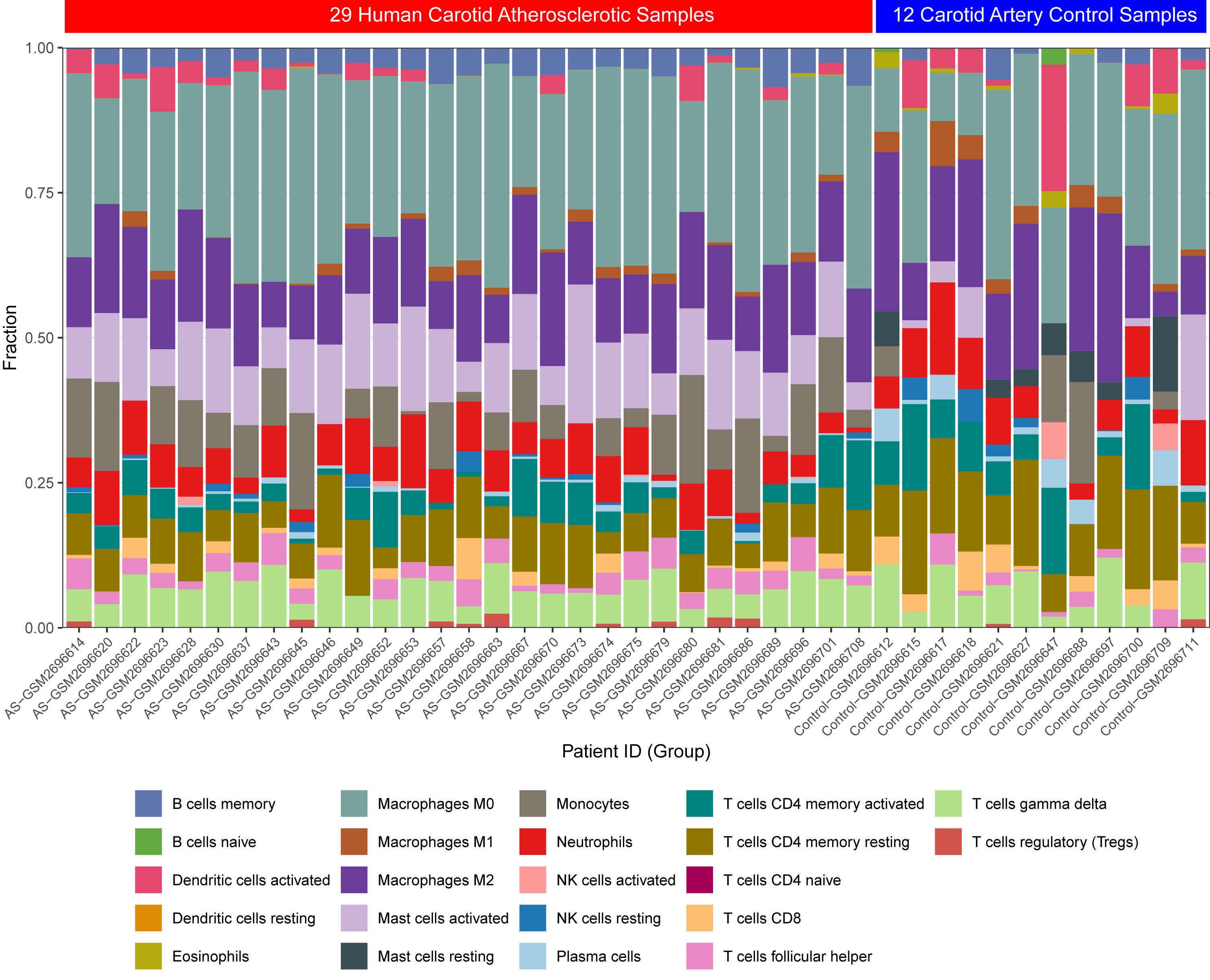

Supplement: Supplementary file 7 [file Image5.tif]
