# Supplementary material for: The role of mitochondria-related genes and immune infiltration in carotid atherosclerosis: identification of hub targets through bioinformatics and machine learning approaches
Source: Front Genet. 2025 Aug 5;16:1597445. doi: 10.3389/fgene.2025.1597445 (PMC12361237; doi:10.3389/fgene.2025.1597445)
Supplement: Supplementary file 3 [file DataSheet1.zip › Supplementary Data/Captions for Supplementary Data.docx]

Supplementary Data

**Supplementary Data 1.** GPL6244-17930

**Supplementary Data 2**. DEGs and associated with pathway enrichments

**Supplementary Data 3**. GO_in modules of various colors
